# Unraveling unique and common cell type-specific mechanisms in glioblastoma multiforme

**DOI:** 10.1016/j.csbj.2021.12.010

**Published:** 2021-12-09

**Authors:** Samreen Fathima, Swati Sinha, Sainitin Donakonda

**Affiliations:** a Department of Biotechnology, Faculty of Life and Allied Health Sciences, MS Ramaiah University of Applied Sciences, Bangalore, India; b Institute of Molecular Immunology and Experimental Oncology, Klinikum Rechts Der Isar, Technical University of Munich, Munich, Germany

**Keywords:** TCGA, The Cancer Genome Atlas, CNS, Cental nervous system, GBM, Glioblastoma multiforme, WHO, World health organization, TP, Primary solid tumour, TR, Recurrent solid tumour, OPC, Oligodendrocyte precursor cell, NFO, Newly formed oligodendrocyte, MO, Myelinating oligodendrocyte, RSEM, RNA-seq by Expectation-Maximization, iDEP, Integrated Differential Expression and Pathway analysis, DEG, Differentially expressed genes, TF, Transcription factor, CAMs, Cell adhesion molecules, INstruct, a database of structurally resolved protein interactome, PDI, Protein domain interactions, PPI, Protein-protein interactions, SIGNOR, Signaling Network Open Resource, PDIN, Protein domain interaction network, GSC, Glioblastoma Stem Cell, NPC, Neural progenitor cell, EMT, Epithelial-mesenchymal transistion, NCBI, National Centre for Biotechnology Information, Glioblastoma multiforme, Glial cell types, Primary solid tumour, Recurrent solid tumour transcription factors, Protein domains, Protein interaction networks

## Abstract

Glioblastoma multiforme persists to be an enigmatic distress in neuro-oncology. Its untethering capacity to thrive in a confined microenvironment, metastasize intracranially, and remain resistant to the systemic treatments, renders this tumour incurable. The glial cell type specificity in GBM remains exploratory. In our study, we aimed to address this problem by studying the GBM at the cell type level in the brain. The cellular makeup of this tumour is composed of genetically altered glial cells which include astrocyte, microglia, oligodendrocyte precursor cell, newly formed oligodendrocyte and myelinating oligodendrocyte. We extracted cell type-specific solid tumour as well as recurrent solid tumour glioma genes, and studied their functional networks and contribution towards gliomagenesis. We identified the principal transcription factors that are found to be regulating vital tumorigenic processes. We also assessed the protein–protein interaction networks at their domain level to get a more microscopic view of the structural and functional operations that transpire in these cells. This yielded the eminent protein regulators exhibiting their regulation in signaling pathways. Overall, our study unveiled regulatory mechanisms in glioma cell types that can be targeted for a more efficient glioma therapy.

## Highlights

• Novel analysis of GBM at its cell type level uncovered underlying mechanisms.
• Various growth-related pathways distinctive to each cell type were revealed.
• Transcription factor network study disclosed chief influencers in each cell type.
• Protein-domain interaction networks identified key regulators.

## Graphical abstract

**Figure:**
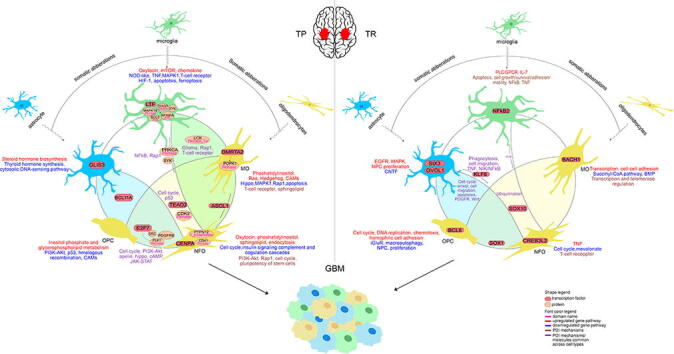

## 1 Introduction

Glial cells are the non-neuronal inhabitants of the central nervous system (CNS) that provide crucial assistance for neural network topology and function [1]. Across the entire territory of the nervous system, glial cells surpass neurons numerically and form a substantial portion of the nervous tissue [2]. Thus, on account of their prominence, dysfunctional glial cells lead to a variety of severe ailments, one of which is the most common form of CNS neoplasm called glioma. Gliomas account for roughly 36% of all primary CNS tumours and almost 80% of all CNS malignant tumours [3], [4]. Glioblastoma multiforme (GBM) is the most common and aggressive (WHO grade IV) form of malignant glioma. This variant ascends from glial cells, either de novo as primary GBM or from pre-existing low-grade astrocytomas as secondary GBM [5]. The conventional approach to treat this form of infiltrative tumor is maximum surgical resection followed by concomitant radiation therapy and chemotherapy using temozolomide [6]. However, its heterogeneity and notorious nature in a difficult-to-access microenvironment, renders this tumour lethal and its complete elimination unattainable. Hence, GBM has a feeble prognosis and relapse is almost inevitable resulting in a median survival rate of 8 to 15 months. Therefore, to successfully treat this tumour, the development of novel therapeutic strategies has emerged as a prime requisite.

In CNS, glial cells are essentially of four main types- astrocyte, oligodendrocyte, microglia and ependymal cell. Astrocytes are the most abundant (approximately 20–40%) irregular star-shaped cell type in the brain [7]. They engage extensively with neurons to provide architectural and metabolic support, and are also crucial for the formation of the blood–brain barrier [8]. Oligodendrocytes have a comparatively lesser amount of cytoplasm [8], and function to insulate axons via enveloping them to form myelin sheaths [9]. Oligodendrocytes are further classified into oligodendrocyte precursor cells (OPCs), premyelinating or newly formed oligodendrocytes (NFOs) and myelinating oligodendrocytes (MOs) based on their degree of differentiation [10], [11]. Microglial cells are the smallest, most eminent immune cells of the CNS that remove debris and account for around 10% of the whole brain cell population [8], [12], [13]. They are the first responders when improper events occur in the brain [12]. Last of all, ependymal cells are ciliated that line the ventricular surface of the CNS and act as the first line of defense [14]. As glial cells are the core constituents of tumour formation and are responsible for maintaining homeostasis in the brain, it becomes imperative to understand and gain insight into their functional operations in GBM. Conversely, it is important to note that even though ependymal cells give rise to a glioma, named ependymoma [15], the involvement of these cells in GBM has inadequate literature support and lacks proper annotation. Thus, we did not consider this cell type in our study.

Although some cancers have well-defined series of events that lead to their genesis, the development of GBM is driven by a complex network of various genetic and molecular perturbations, resulting in critical changes in signaling pathways [16]. For example, EGFR signaling pathway [17], Ras pathway [18], PTEN signaling pathways[19], retinoblastoma pathway [20], etc. are some of the vital signaling pathways that undergo modification in GBM. Most of these unprecedented discoveries have transpired at the tissue level analysis of the tumour. However, little is known about the GBM formation at the level of aforementioned cell types. This consequently opened a gateway to direct our focus on the functional alterations and contributions made independently by each glial cell type in GBM.

In this study, we conducted an analysis on differential gene expression profiles of GBM primary solid tumour (TP) and recurrent solid tumour (TR) in astrocytes, microglial cells, OPCs, NFOs, and MOs. Our investigation enabled us to dissect top transcription factor regulators and scrutinize protein-domain level network interactions in each of these GBM TP and TR specific cell types. This allowed us to make some noteworthy observations, and revealed common as well as unique mechanisms that occur in these glial cells. These findings can be further validated to inspect their relevance as targets for drug therapy in the hope of a sustainable treatment against GBM.

## 2 Materials and methods

### 2.1 RNA-sequencing data analysis of GBM and data acquisition of brain cell type-specific genes

The Cancer Genome Atlas (TCGA) firehouse (https://gdac.broadinstitute.org/) provided us with RNA-Seq by Expectation-Maximization (RSEM) normalized expression data for normal brain and TP, TR cancer from GBM patients. The DESeq2 package, which is part of the integrated Differential Expression and Pathway analysis (iDEP v.90) software [21], was used to identify the differentially expressed genes (DEGs) using the normalized expression data. Genes that met the following criteria were considered as DEG: *p-*adjusted-value ≤ 0.05 and log_2_ fold change 1 (absolute fold change: 2) [22]. The bulk RNA and single-cell sequencing gene datasets of normal brain astrocyte, microglia, OPC and NFO were collected from the literature [23], [24]. Normalized expression data related to OPC, NFO and MO were downloaded from GEO database (accession ID: GSE52564) [23] and DEGs were extracted using iDEP v.90 software [21]. In these, the bulk RNA sequenced mouse genes [23] of each brain cell type were mapped to their human orthologs by employing g:Profiler (https://biit.cs.ut.ee/gprofiler/orth). Ultimately, we went ahead with the genes that were available in human organism and performed our investigation.

### 2.2 Overlap analysis to retrieve cell type-specific glioma gene datasets

We mined 1388 astrocyte, 1300 microglia, 2538 OPC, 4118 NFO and 2745 MO genes [23], [24], respectively. We overlapped these sets of genes with the DEGs of GBM TP and TR to extract subsets of unique and common cell type-specific glioma genes. We considered these genes for further analysis.

### 2.3 Transcription factor network analysis

We curated transcription factors (TFs) from the above-mentioned subsets of genes by using the human TF atlas v1.01 (http://humantfs.ccbr.utoronto.ca/), an index of 1639 known and probable TFs [25]. We overlapped these TFs with glioma cell type-specific DEGs and extracted the common TFs. They were used to compute the TF co-regulatory target network using CoRegNet, a R/Bioconductor package [26]. This package administers the h-LICORN algorithm [27], [28] to reconstruct a network by identifying experimentally validated co-regulators and co-inhibitors for a given set of gene expression data. To procure a more refined network, input features such as TF-gene interaction data, (e.g., ChIP-sequencing, TF-binding site), to endorse the regulatory interactions were incorporated as additional evidence. Furthermore, this package also implements a function to evaluate the TF activity by measuring their transcriptional influence. This influence is estimated in a sample-specific fashion based on a comparison of the expression of the activated and repressed targets of a regulator, and it does so while being more noise-resistant than the standard network reconstruction approaches [26]. We further investigated the association of two or more TFs by conducting a TF-TF correlation network analysis. This was done by generating a table of correlation coefficients and their corresponding p-values using the Hmisc package (https://hbiostat.org/R/Hmisc/). We then formatted the correlation matrix that consists of the cormat matrix of the correlation coefficients, and the pmat matrix of the correlation p-values, by using the function flattenCorrMatrix. The TF co-regulatory and TF-TF correlation networks were visualized in Cytoscape v3.8.0 [29].

### 2.4 Protein domain interaction network analysis

We extracted the human protein domain interactions (PDIs) data from the INstruct database (http://instruct.yulab.org), a 3-dimensional structurally resolved library of high-quality protein interactome networks containing 6585 interactions at protein domain level. INstruct consists of interactions that were derived from some of the most prominent interaction databases and screened to present only binary interactions that met its stringent quality standards [30]. To visualize the protein domain interaction network (PDIN) of the retrieved PDI INstruct data, SIGNOR v.2.0 (http://signor.uniroma2.it), the SIGnaling Network Open Resource [31] was used. SIGNOR is a compilation of experimentally validated causal relationships, i.e., interactions in which a source entity has an influence on a target entity (e.g. activation, inhibition, binding). Proteins having the highest number of interactions were considered as top regulators.

### 2.5 Functional pathway enrichment analysis

We used METASCAPE [32] to undertake pathway analysis of DEGs, TFs, and proteins from PDI networks. These included KEGG pathways and gene ontology biological process. Using a p-adjusted value ≤ 0.05, the pathways were deemed statistically significant. Due to the unavailability of enriched METASCAPE data for a few DEGs and PDI proteins, we derived their function and pathway information from the National Centre for Biotechnology Information (NCBI) [33] and UniProt [34].

### 2.6 Data visualization and statistical analysis

In this study, we used R statistical software v3.6.3 (https://www.r-project.org/) to conduct data visualization and statistical analysis. Hierarchical clustering analysis was done using the Euclidean similarity metric and visualized as dendrogram using factoextra R package. Volcano plots illustrating the top DEGs in and across glioma-specific cell types were generated using the ggplot2, ggrepel, EnhancedVolcano, gghighlight, and dplyr R packages. The differential expression analysis of GBM and the top protein regulators in PDINs were visualized as barplots using the ggpubr R package that implemented the ggpbarplot function. Unique and common glioma-specific genes/TFs in cell types were pictured as barplots using the UpSet R package. The Pheatmap R package was used to render the heatmaps.

## 3 Results

### 3.1 Workflow for dissecting unique and common mechanisms in GBM at the single-cell level

**Fig. 1:**
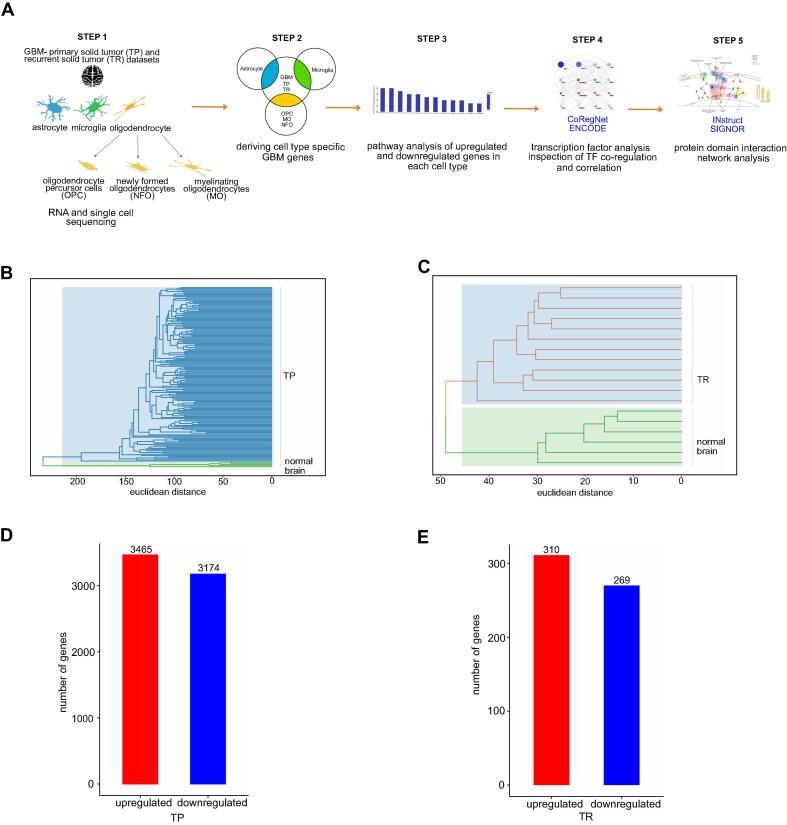
**RNA-sequencing analysis of GBM.** (**A**) Schematic representation of acquiring cell type-specific GBM data and the consolidative analyses. (**B-C**) GBM hierarchical clustering analysis of normal brain, TP and normal brain, TR cancer patients, respectively. (**D-E**) Differential gene expression analysis of GBM TP and TR.

GBM is widely studied at tissue level, however, here we scrutinized the events that occur in the cell types of this tumour at its primary as well as recurrent stages. The workflow we followed in this study is briefly illustrated as a pipeline in Fig. 1**A**. To begin with, we extracted the RNA sequencing gene expression dataset of GBM; normal brain, TP and TR cancer patients, from The Cancer Genome Atlas (TCGA). The bulk RNA and single-cell sequencing gene datasets of the brain cell types- astrocyte, microglia, OPC, NFO and MO- were mined from Zhang Y et al [23] and Fan X et al [24]. Next, we culled out cell type-specific glioma genes and conducted the pathway enrichment analysis for these differentially expressed genes (DEGs). Furthermore, transcription factor (TF) analysis was implemented wherein we constructed the TF-gene co-regulatory and TF-TF correlation networks. Finally, we performed the protein domain interaction network (PDIN) analysis which gave us a detailed landscape of the mechanisms that occur at the proteomic level in each GBM cell type.

### 3.2 Transcriptomic analysis of GBM and derivation of cell type-specific glioma genes

To elucidate the functional dynamics that occur due to the gene expression in GBM, we performed the transcriptomic examination. The hierarchical clustering analysis of GBM TP and TR expression profiles evidently showed clear partitioning of the normal brain and cancer patient samples (Fig. 1**B-C)**. We detected 6639 DEGs in GBM TP, out of which 3465 were upregulated and 3174 were downregulated (Fig. 1**D)**. In GBM TR, we identified 579 DEGs, out of which 310 were upregulated and 269 were downregulated (Fig. 1**E).**

**Fig. 2:**
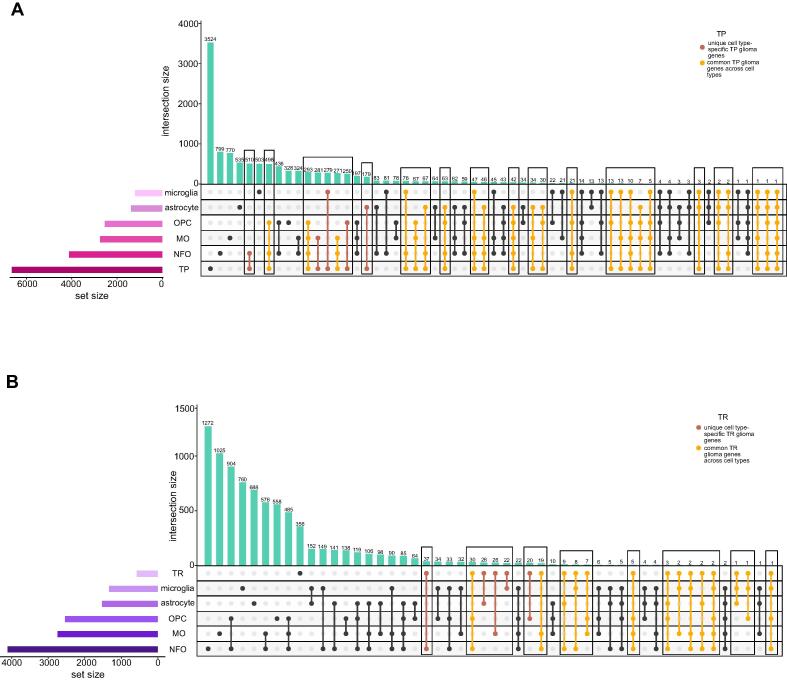
Unique and common glial cell type-specific glioma genes. (**A-B**) The UpSet plot displaying the glioma-specific unique and common genes across cell types in TP and TR.

Our next step involved overlapping of the GBM TP, TR, astrocyte, microglia, OPC, NFO and MO gene datasets in order to derive cell type-specific glioma genes. This allowed us to cull down subsets of unique cell type-specific glioma genes and common glioma genes across the cell types as shown in Fig. 2**A-B**.

**Fig. 3:**
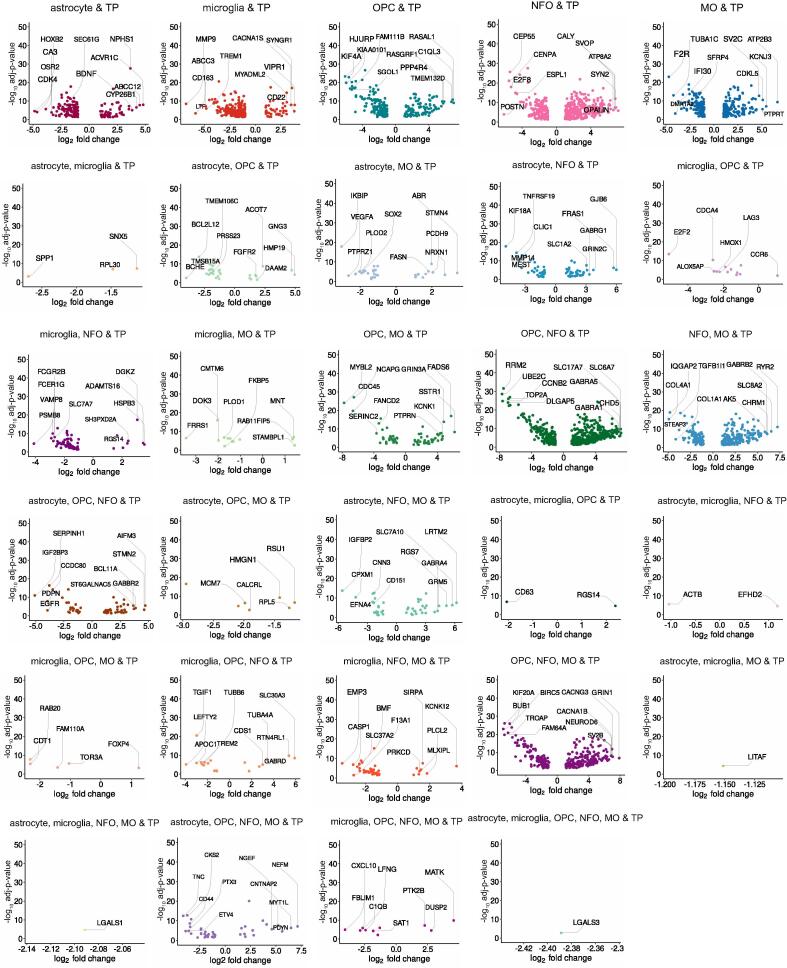
Top TP DEGs unique and common across cell types. The volcano plots exhibiting top upregulated and downregulated genes between TP glioma and cell types.

**Fig. 4:**
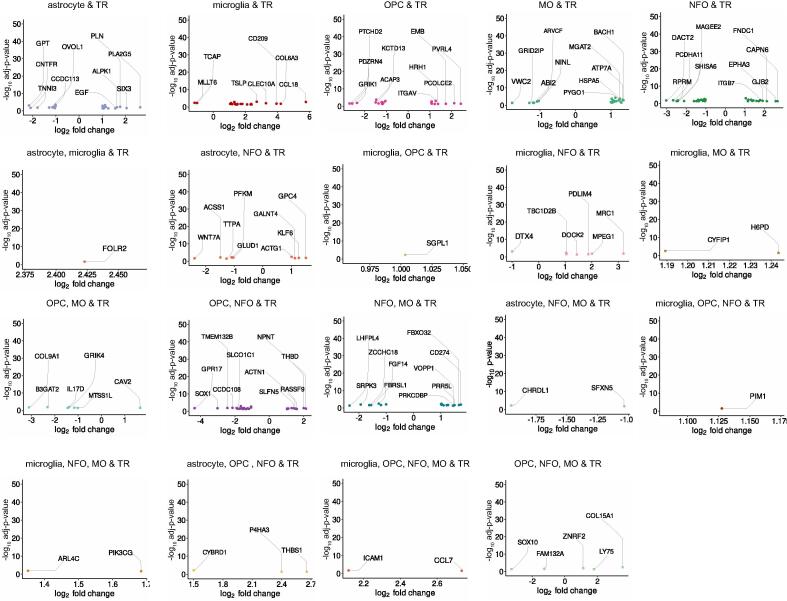
Top TR DEGs unique and common across cell types. The volcano plots exhibiting top upregulated and downregulated genes between TR glioma and cell types.

In total, we obtained 29 subsets of TP glioma genes and 19 subsets of TR glioma genes. These include genes that are unique to the cell types and common across various combinations of these cell types. The genes in each subset were categorized based on their differential expression (Supplementary Table S1A-AC and S2A-S). The DEGs are illustrated in Fig. 3 and Fig. 4 with the top upregulated and downregulated genes highlighted. In conclusion, we winnowed down glioma genes specific to glial cell types and considered these genes in our further analysis.

### 3.3 Signaling pathways enriched in glioma-specific cell types

To enhance our knowledge on the involvement of these cell-specific DEGs in GBM, we performed pathway enrichment analysis of upregulated and downregulated genes separately in TP and TR. These analyses revealed that in TP astrocytes, genes participate in hormone synthesis and metabolism (Supplementary Table S3A). In TP microglia, genes are engaged in signaling and cell death pathways (Supplementary Table S3B). In TP OPC, they regulate metabolism and signaling pathways (Supplementary Table S3C). In TP NFO, genes are involved in lipid signaling and coagulation cascades (Supplementary Table S3D). Lastly, in TP MO, the upregulated genes are linked to signaling pathways and metabolism (Supplementary Table S3E).

Correspondingly, in TR astrocyte, genes are involved in signaling cascades (Supplementary Table S4A). In TR microglia, genes take part in signaling pathways (Supplementary Table S4B). In TR OPC, gene are linked to cell cycle and signaling pathways (Supplementary Table S4C). In TR NFO, genes are engaged in cell cycle and TNF signaling pathway (Supplementary Table S4D). Finally, in TR MO, they participate in cell adhesion and immune related pathways (Supplementary Table S4E).

The pathway enrichment analysis was also conducted for genes that were common between the cell types and GBM. In sum, the common TP upregulated genes are involved in signaling, cell adhesion and metabolic pathways (Supplementary Table S3F-AC). Similarly, the common TP downregulated genes are involved in cell growth, tumor progression and T-cell regulation (Supplementary Table S3F-AC).

Furthermore, the TR upregulated genes that are common across different combinations of cell types participate in signaling pathways, cell growth, apoptosis, and immune response (Supplementary Table S4 F-S). Likewise, the common TR downregulated genes participate in signaling pathways such as metabolism, cell migration and adhesion (Supplementary Table S4 F-S). In a nutshell, this analysis revealed that although the DEGs in these cancer cell types participate in different signaling pathways, they are common in growth-related functions.

### 3.4 Identification of unique and common transcription factors in each cell type

**Fig. 5:**
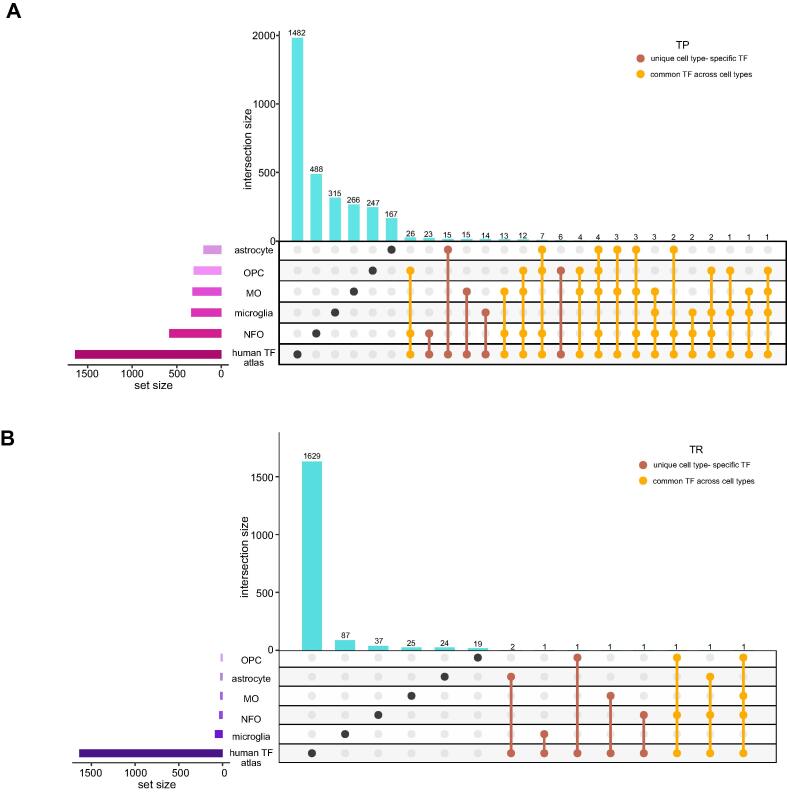
Unique and common TFs in glioma cell types. **(A-B)** The UpSet plots represent TP and TR transcription factors (TFs) unique and common in astrocyte, microglia, OPC, NFO and MO.

Transcription factors (TFs) play a critical role in the regulation of gene transcription and expression processes. This corresponds as a vital element in an intricate network system that governs healthy cell development and function [35]. Hence, owing to their significance, we investigated the TFs present in the GBM-specific brain cell types. Primarily, we filtered the TFs from the gene datasets by performing an overlap analysis with the Human TF atlas catalog containing 1639 recognized and likely human TFs [25]. This enabled us to segregate out cell type-specific TP and TR glioma TFs that are unique and common across astrocyte, microglia, OPC, NFO and MO (Fig. 5A-B) (Supplementary Table S5A-T and S6A-H). This provided us with 20 subsets of TFs in TP and 8 subsets of TFs in TR. The 8 subsets in TR include TFs in astrocyte (n = 2), microglia (n = 1), OPC (n = 1), NFO (n = 1), MO (n = 1), OPC and NFO (n = 1), astrocyte and NFO (n = 1), and lastly in OPC, NFO and MO (n = 1). However, in our further network analysis, we only considered TFs in TP and not the TR TFs, since they were very less in number; TF-target network cannot be constructed in TR dataset.

We initiated our examination with putative TF-target co-regulation analysis. This was done via CoRegNet that enriched the networks with the ENCODE ChIP-sequencing data as additional evidence [26]. Out of 20 TF subsets in TP, this analysis gave results for only 8 TF subsets due to numerically less TFs in the remaining subsets. In TP astrocytes, we observed a total of 15 TFs and 9 of them are enriched with ChIP-seq data (Supplementary Fig. S1**A)**. In TP microglia, out of 14 total TFs, 11 are complemented with ChIP-seq (Supplementary Fig. S1**B).** In TP MO, out of a total of 15 TFs, 11 were supported by ChIP-seq data (Supplementary Fig. S1**C)**. In TP NFO, out of a total of 23 TFs, 13 are corroborated with the ChIP-seq data (Supplementary Fig. S1**D)**. In TP OPC and NFO, out of a total of 26 TFs, 16 were complemented with ChIP-Seq data (Supplementary Fig. S1**E).** In TFs common across TP NFO and MO, a total of 13 were observed out of which 8 were enriched with ChIP-Seq data (Supplementary Fig. S1**F).** In TP astrocyte, OPC and NFO, a total of 7 TFs were found, out of which 4 were supported with ChIP-Seq data (Supplementary Fig. S1**G)**. Lastly, in TP OPC, NFO and MO, a total of 12 TFs were identified, out of which 8 were corroborated with ChIP-Seq data (Supplementary Fig. SH). Finally, we observed unique novel putative TFs in TP astrocyte (n = 6), microglia (n = 2), MO (n = 4) and NFO (n = 10) (Supplementary Fig. S2**A-D).** The common novel putative TFs were also observed in TP OPC and NFO (n = 10), NFO and MO (n = 5), astrocyte, OPC and NFO (n = 3) and OPC, NFO and MO (n = 4) (Supplementary Fig. S2**E-H).** For these novel TFs we did not find evidences in ChIP-seq datasets from ENCODE. Overall, this analysis revealed enriched common and unique transcription factors in glioma-specific cell types.

### 3.5 Transcription factor co-regulatory network analysis revealed critical TFs

**Fig. 6:**
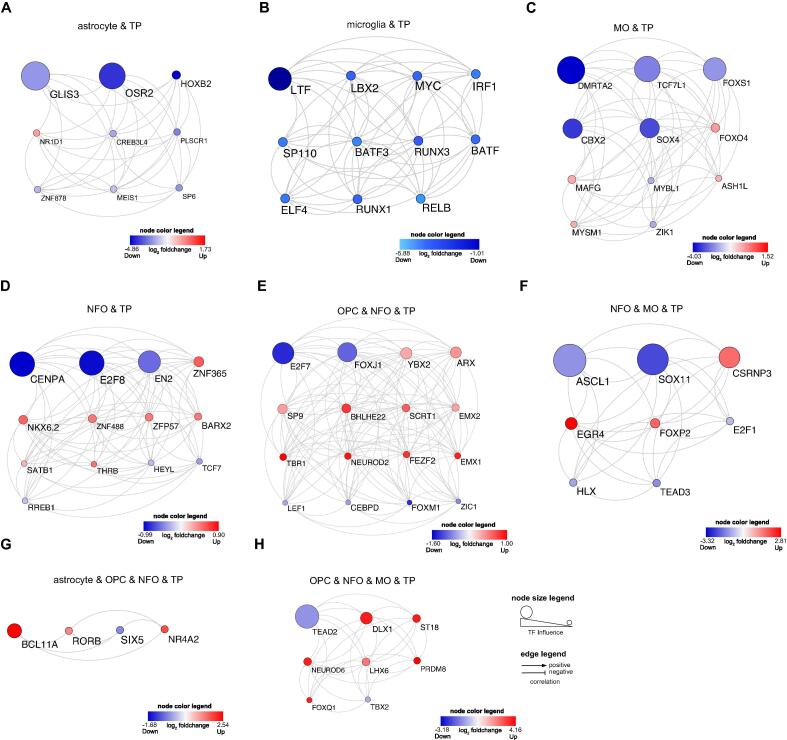
Transcription factor network analysis in TP GBM cell types. (**A-H**) TF-TF correlation networks displaying hierarchical transcriptional influence of TFs. Note: Red and blue indicate up and down regulation, respectively.

To gain a deeper understanding of the functionality and association between the TFs, we conducted a TF-TF correlation analysis by computing the correlation coefficient to generate networks displaying positive (activation) and negative (inhibition) interactions. We further refined this investigation by conducting TF transcriptional influence activity analysis using the CoRegNet package [26], this indicates how many targets are regulated by each TF. Positive influence indicates that TF is regulating upregulated targets and negative influence denotes that TF is regulating downregulated targets. The transcriptional influence is computed for the TFs with a suitable number of targets in the transcriptional network (minimum 10 activated and 10 repressed). This further narrowed down the known TFs and gave us the top regulators. In TP astrocyte, we observed 9 TFs with GLIS3 being the most significant TF influencer followed by OSR2 and HOXB2 (Fig. 6**A)**. Similarly, in TP microglia, correlation between 11 TFs was seen, with LTF being the prominent TF (Fig. 6**B)**. In TP MO, correlation between 11 TFs was found, with DMRTA2 being the notable TF influencer, followed by TCF7L1and FOXS1 (Fig. 6**C)**. In TP NFO, correlation between 13 TFs was noted, with CENPA being the influential TF followed by E2F8 and EN2 (Fig. 6**D)**. In OPC and NFO, correlation between 16 TFs was detected with E2F7 being the eminent TF influencer followed by FOXJ1 (Fig. 6**E)**. Similarly, in TFs common between NFO and MO, correlation between 8 TFs was witnessed, with ASCL1 being the principal TF, followed by SOX11 and CSRNP3 (Fig. 6**F)**. In OPC, NFO and MO, correlation between 8 TFs was observed, with TEAD2 being the chief TF followed by DLX1 and ST18 (Fig. 6**G)**. Lastly, in astrocyte, OPC and NFO, correlation between 4 TFs was seen, with BCL11A being the salient TF influencer (Fig. 6**H)**. In sum, TF-TF network analysis revealed top TF regulators in each glioma-specific cell type.

### 3.6 Transcription factors in each cell type regulate various signaling and metabolic processes

**Fig. 7:**
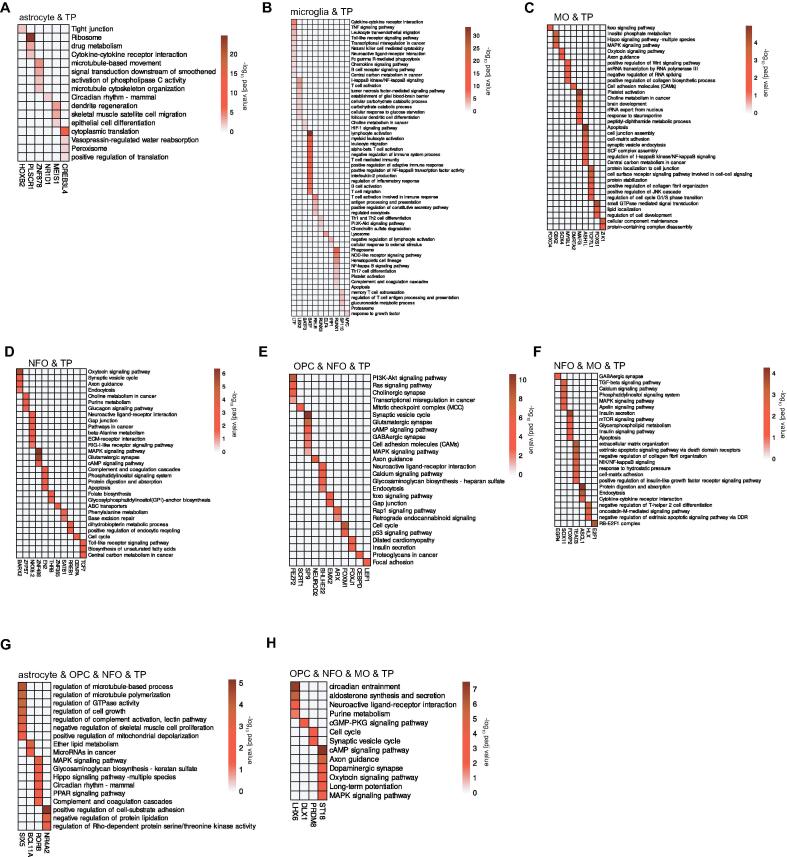
Pathway enrichment analysis of transcription factors. (**A-H**) The heatmaps depict the most significant (P ≤ 0.05) pathways in and across TP astrocyte, microglia, OPC, NFO and MO regulated by the TFs.

Additionally, to better acquire knowledge of the processes the TFs participate in each of the cell types, we performed a pathway enrichment analysis. This evaluation revealed that in TP astrocyte, TFs ZNF878 and CREB3L4 are involved in highest number of processes such as signal transduction downstream of smoothened protein which is involved in hedgehog signaling [36], activation of phospholipase C activity and peroxisome, positive regulation of translation, respectively (Fig. 7**A)**. Consequently, in TP microglia, LTF, the significant TF regulator is found to regulate signaling pathways like TNF, TLR, chemokine, B-cell receptor and processes such as transcriptional misregulation, natural killer cell mediated cytotoxicity, phagocytosis and carbon metabolism in cancer (Fig. 7**B)**. Moreover, the TF BATF is found to significantly regulate the highest number of processes such as negative regulation of immune system, positive regulation of adaptive immune response and NFκB TF activity, B cell activation, T cell migration etc. In TP MO, the top regulator, DMRTA2, is seen to be involved in CAMs (Fig. 7**C)**. In TP NFO, the eminent regulator, CENPA, is observed to be involved in cell cycle whereas the third top regulator, EN2, is found to be involved in apoptosis, phosphatidylinositol signaling system, complement and coagulation cascades. However, NKX6.2 is seen to participate in the highest number of processes like pathways in cancer, beta-alanine metabolism, RIG-I-like signaling pathway etc. (Fig. 7**D)**. In addition, ASH1L is seen to regulate the highest number of processes such as apoptosis, NFκB signaling pathway, carbon metabolism in cancer etc. Similarly, in TP OPC and NFO, the TF SP9 is witnessed to significantly regulate highest number of processes such as CAMs, and cAMP, MAPK signaling pathways (Fig. 7**E)**.

Furthermore, in TFs common between TP NFO and MO, the prominent TF regulator, ASCL1, is found to be involved in endocytosis and cytokine-cytokine receptor interaction, whereas the second top TF influencer, SOX11, is found to regulate signaling pathways such as TGF-β, MAPK, calcium, phosphatidylinositol and apelin (Fig. 7**F)**. Additionally, the TF TEAD3 is found to regulate highest number of signaling pathways such as extrinsic apoptotic via death domain receptors, NFκB and insulin-like growth factor receptor. In TP astrocyte, OPC and NFO, the notable TF, BCL11A, is involved in microRNAs in cancer, whereas the TF SIX5 is seen to regulate highest number of processes such as regulation of GTPase activity, cell growth, complement activation, lectin pathway etc (Fig. 7**G)**. Finally, In TP OPC, NFO and MO, the second most significant TF influencer DLX1, is noted to be involved in cGMP-PKG signaling pathway and the third top TF influencer, ST18, is noted to be involved in the highest number of processes that include cAMP, oxytocin and MAPK signaling pathways (Fig. 7**H)**. Taken together, the TF network pathway analysis gave us a thorough view of the important cancer-related pathways they regulate in each glioma-specific cell type.

### 3.7 Protein-protein interaction network analysis at the domain level

Protein-protein interactions (PPIs) are the fundamental driving force behind the working of a complex network of processes. The interactions occur when the respective domains of each protein physically associate with each other, thereby corresponding to facilitate a particular function[37]. Hence, studying PPIs of GBM at the protein domain level gives a more holistic view of the functional operations that materialize in each cell type. We began by enrolling the TP and TR genes into the INstruct database and retrieved high-quality protein-domain interaction data (Supplementary Table S7A-X and S8A-I). This data was further logged into the SIGnaling Network Open Resource (SIGNOR 2.0) database to visualize the regulatoryprotein-domain interaction networks (PDINs). Lastly, we also conducted pathway enrichment analysis for these proteins.

**Fig. 8:**
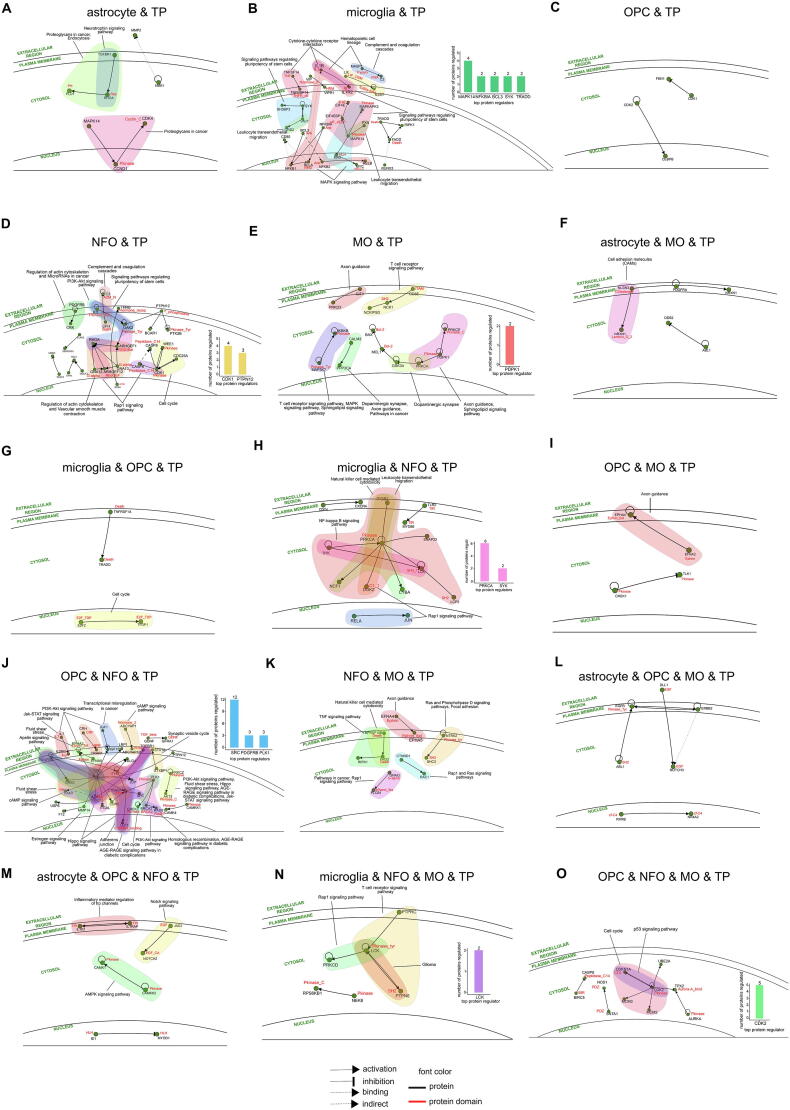
Protein-domain interaction network (PDIN) analysis of TP proteins in cell types. (**A-O**) Comprehensive visualization of PDINs in each cell type and the most significant (Padj ≤ 0.05) pathways they participate in. The bar plots illustrate the top protein regulators and the number of proteins they regulate in astrocyte, microglia, OPC, NFO and MO.

In TP astrocyte, some important processes like neurotrophin signaling pathway and endocytosis were seen (Fig. 8**A)**. In TP microglia, we observed signaling pathways that regulate pluripotency of stem cell, MAPK signaling pathway, complement and coagulation cascades as some of the important processes (Fig. 8**B)**. We further winnowed down MAPK14 (domain- Pkinase), NFKBIA (domain- Ank), BCL3 (domain- Ank), SYK and TRADD (domain- Death) as the significant protein regulators that are regulating 4, 2, 2, 2 and 2 proteins, respectively. The top TF influencer LTF, is found to regulate BCL3. In TP OPC, the participation of the PDIs in pathways and processes needs to be studied further (Fig. 8**C)**. In TP NFO, the PDIs were seen to take part in processes such as cell cycle, signaling pathways regulating pluripotency of stem cells, Rap1 and PI3K-Akt signaling pathways, complement and coagulation cascades (Fig. 8**D)**. The two most prominent protein regulators were found to be CDK1 (domain- Pkinase) and PTPN12 (domain- Y_phosphatase) regulating 4 and 3 proteins, respectively. In TP MO, we witnessed PDIs engaging in signaling pathways such as T-cell receptor, MAPK, and sphingolipid (Fig. 8**E)**. In this PDIN, PDPK1 (domain- Pkinase) was found to be the chief protein regulator that is regulating 2 proteins. PDPK1 is regulated by the most significant TF influencer DMRTA2 in this cell type.

Similarly, this analysis was also conducted for TP proteins common across cell types. In TP astrocyte and MO, the PDI was found to participate in CAMs process (Fig. 8**F)**. In TP microglia and OPC, a PDI is observed to engage in cell cycle (Fig. 8**G)**. In TP microglia and NFO, the vital processes witnessed were Rap1, NFκB signaling pathways and natural killer cell mediated cytotoxicity (Fig. 8**H)**. PRKCA (domain- Pkinase) and SYK were noted to be the influential protein regulators regulating 6 and 2 proteins, respectively. In TP OPC and MO, a PDI takes part in axon guidance (Fig. 8**I)**. In TP OPC and NFO, vital processes like cell cycle, transcriptional misregulation, fluid sheer stress and signaling pathways such as Jak-STAT, hippo, PI3K-Akt, AGE-RAGE, apelin, and cAMP were observed (Fig. 8**J)**. The 3 notable protein regulators were winnowed down to SRC, PDGFRB and PLK1 (domain- Pkinase) that are regulating 12, 3 and 3 proteins, respectively. PLK1 was found to be governed by the top TF influencer i.e. E2F7. In TP NFO and MO, the PDIs participate in Ras, Rap1, TNF, phospholipase D signaling pathways and natural killer cell mediated cytotoxicity (Fig. 8**K**). In TP astrocyte, OPC and MO, we noted the PDIs but their involvement in processes needs further evaluation (Fig. 8**L)**. In TP astrocyte, OPC and NFO, the PDIs were observed to take part in Notch and AMPK signaling pathways (Fig. 8**M**). In TP microglia, NFO and MO, T-cell receptor and Rap1 signaling pathways were seen with LCK (domain- Pkinase_tyr) being the principal protein regulator governing 2 proteins (Fig. 8**N)**. Lastly, in TP OPC, NFO and MO, the PDIs are involved in cell cycle and p53 signaling pathway with CDK2 (domain- Pkinase) as the salient protein regulator governing 5 proteins (Fig. 8**O)**.

**Fig. 9:**
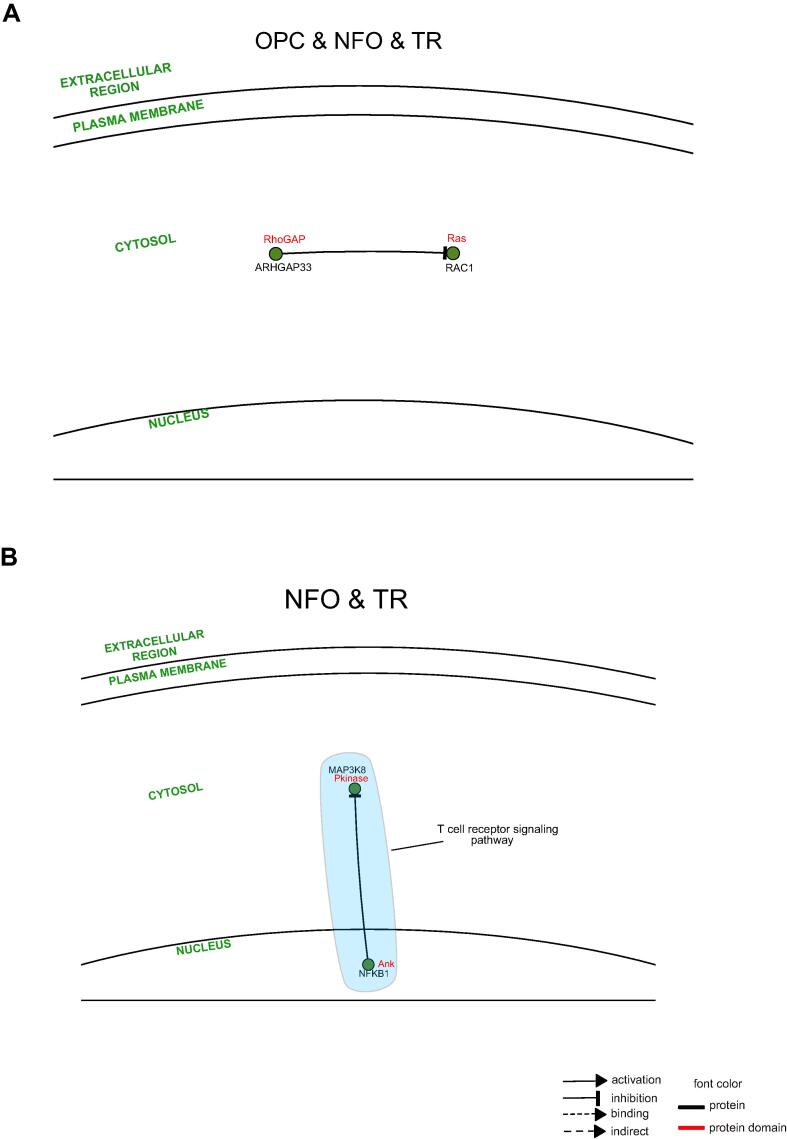
Protein-domain interaction network (PDIN) analysis of TR proteins in cell types. (**A-B**) Comprehensive visualization of PDINs in each cell type and the most significant (Padj ≤ 0.05) pathways they participate in.

This analysis was similarly conducted on TR cell types. However, the SIGNOR result was available for only TR NFO, and TR OPC and NFO proteins. In TR OPC and NFO, the participation of the PDI needs further examination (Fig. 9**A)**. Lastly, in TR NFO, the PDI is found to take part in T-cell receptor signaling pathway (Fig. 9**B)**.

Overall, this analysis manifested a comprehensive view of the domain-level interactions of the proteins, their regulation, and the pathways they control.

**Fig. 10:**
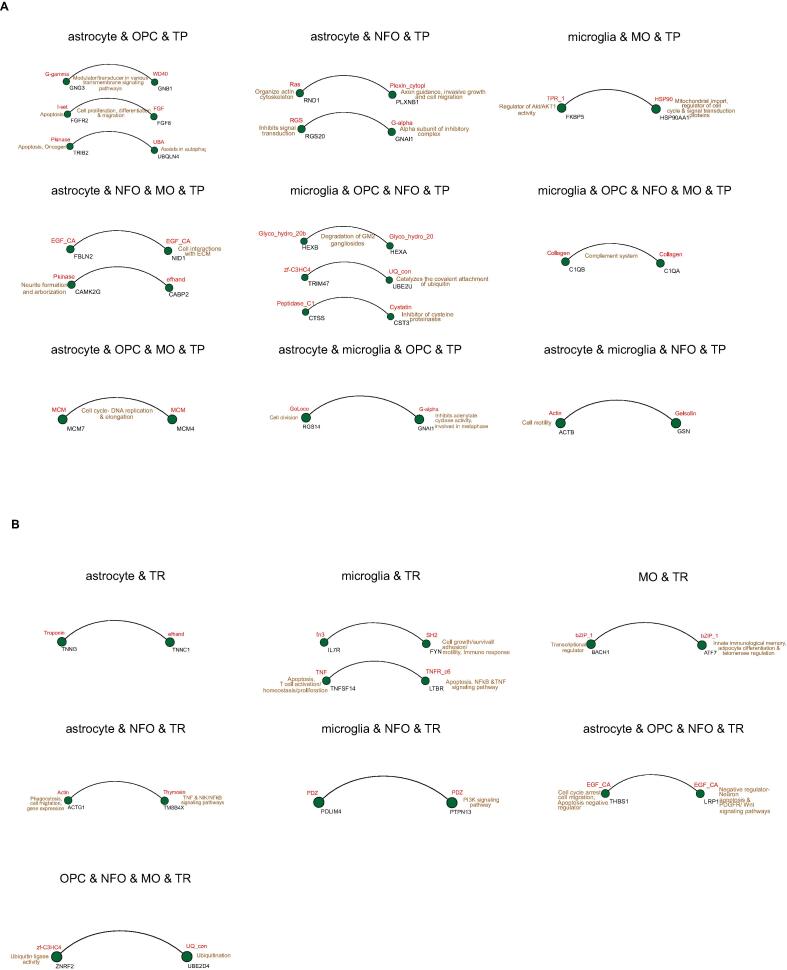
Protein domain interactions (PDIs) between cell types. (**A-B**) PDIs in and across cell types with their functions in TP and TR, respectively.

For some proteins, the SIGNOR visualization and functional funct data from enrichment analysis was unobtainable. However, we found that these How shared PDIs between TP astrocyte and OPC participate in cell proliferation, differentiation, migration, apoptosis, oncogenesis, act as modulator/transducer in various transmembrane signaling pathways and assists in autophagy. The PDIs common between TP astrocyte and NFO take part in processes such as invasive growth, cell migration and signal transduction inhibition. In TP microglia and MO, the PDI is involved in regulation of Akt/AKT1 activity, mitochondrial import, regulation of cell cycle and signal transduction proteins. The PDIs in TP astrocyte, NFO and MO are involved in cell interaction with ECM, neurite formation and arborization. In TP microglia, OPC and NFO, the PDIs take part in catalyzing the covalent attachment of ubiquitin and inhibition of cysteine proteinases. The PDI shared between microglia, OPC, NFO and MO, is engaged in the complement system. The PDI between TP astrocyte, microglia and OPC, takes part in cell division and inhibits adenylate cyclase activity. The common PDI between astrocyte, OPC and MO is involved in cell cycle, DNA replication and elongation. Lastly, the mutual PDI among astrocyte, microglia and NFO takes part in the cell motility process (Fig. 10**A)**.

Similarly, the PDIs in TR microglia takes part in cell growth, survival, motility, immune response, apoptosis, T cell activation, homeostasis, proliferation and signaling pathways such as NFκB and TNF. The TR MO PDI participates in innate immunological memory and telomerase regulation. PDI shared between TR astrocyte and NFO is involved in phagocytosis, cell migration, gene expression, TNF and NFκB signaling pathways. The PDI in TR microglia and NFO engages in PI3K signaling pathway whereas mutual PDI between TR astrocyte, OPC and NFO, is involved in cell cycle arrest, cell migration, negative regulation of apoptosis, PDGFR and Wnt signaling pathways. Finally, the PDI common between TR OPC, NFO and MO engages in ubiquitination. (Fig. 10**B)**.

Taken together, this analysis revealed the key protein domain interactions and functions regulated in GBM TP and TR by proteins across cell types.

### 3.8 Prospective gliomagenesis model of GBM at its cell type level

**Fig.11:**
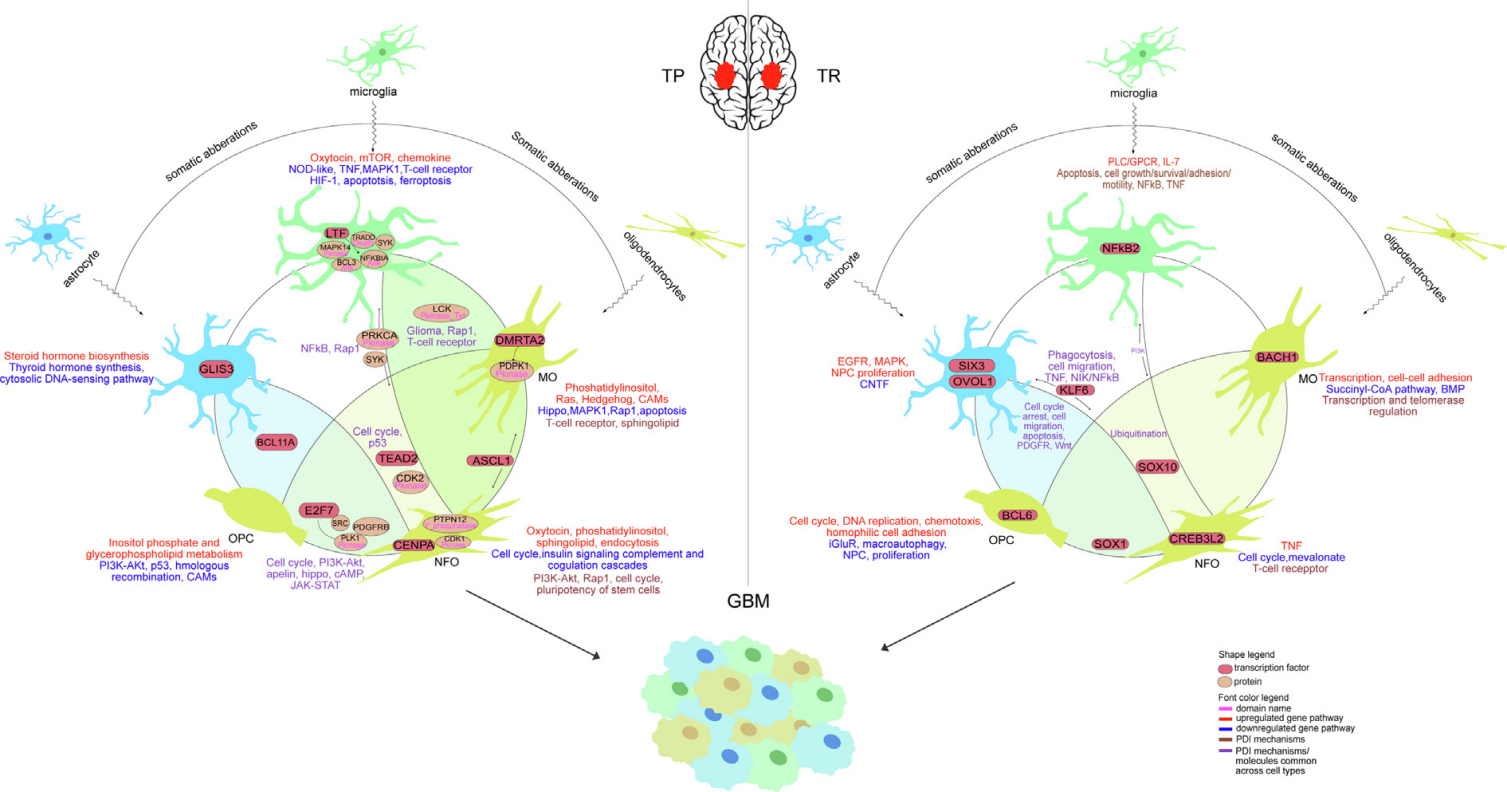
**Graphical abstract of our findings.** Depiction of prospective gliomagenesis model in GBM at its cell type level.

Somatic aberrations induce the transformation of healthy glial cells into cancerous cells. Our findings give crucial insight into the various unique and common molecular mechanisms that undergo alterations in the glial cell types leading to the inception of GBM (Fig. 11). The differentially expressed pathways in astrocyte, microglia, OPC, NFO, and MO were found in TP as well as TR GBM. Our integrated transcriptional and proteomic analyses reveal critical transcription factors and proteins in each and across all five cell types that can be studied and validated as drug targets to treat GBM.

## 4 Discussion

Due to its heterogenic nature and ability to evade any form of therapy regimens, GBM remains to be the most invasive and detrimental brain tumour. Designing a compelling treatment strategy against this lethal glioma has been a challenging issue globally. The studies being conducted are maximally based and focused on the tumour tissue as a whole. This prompted us to renew the standard approach and perform an extensive in-silico analysis at the cell type level.

Glial cells or neuroglia are the non-excitable group of support cells in the nervous system that predominate the neurons in abundance. The CNS consists of astrocyte, microglia, and oligodendrocytes [38]. Oligodendrocytes are further divided into OPC, NFO and MO. Before myelination occurs, the OPCs first convert into NFOs and then finally into mature MOs that myelinate the axons [39], [40]. Malfunction of these glial cells consequently gives rise to gliomas. For example OPCs have been widely studied for their contribution in gliomas [41], [42]. The transcription factors OLIG2 and SOX10 are expressed in all cells of the oligodendrocyte lineage [10], and these TFs exhibit broad expression across the glioma subtypes [43], [44].

In this study, we retrieved the gene expression dataset of GBM; normal brain, TP and TR cancer patients (Fig. 1**B-E)**. We compared this dataset with the gene sets from bulk RNA and single-cell sequencing datasets of astrocyte, microglia, OPC, NFO and MO. This winnowed down subsets of TP and TR glioma genes that are unique as well as common to the cell types (Fig. 2**A-B)**. Differential expression analysis allowed us to segregate upregulated and downregulated genes (Supplementary Table 1A-AC and 2A-S). Additionally, we highlighted the top DEGs in each of these subsets (Fig. 3, Fig. 4). Further investigation of these single-cell specific TP GBM genes revealed signaling pathways such as oxytocin, chemokine, mTOR, phosphatidylinositol, sphingolipid, Ras, hedgehog and mechanisms such as steroid hormone biosynthesis, inositol phosphate, glycerophospholipid, purine metabolism, endocytosis choline metabolism in cancer, CAMs etc. to be governed by the upregulated genes unique to the cell types. In contrast, the downregulated genes unique to the cell types are involved in signaling pathways such as NOD-like, TNF, T-cell receptor, MAPK, HIF-1, p53, PI3K-Akt, insulin, hippo, Rap1 and mechanisms such as thyroid hormone synthesis, cytosolic DNA-sensing pathway, apoptosis, ferroptosis, transcriptional misregulation in cancer, natural killer cell mediated cytotoxicity, central carbon metabolism in cancer, homologous recombination, pyrimidine and gluthathione metabolism, CAMs etc.

Our analysis also disclosed the pathways that are employed by the genes common across various combinations of cell types. This revealed that the common TP upregulated genes are involved in signaling pathways such as cAMP, hippo, apelin, MAPK, adipocytokine, calcium, phospholipase D, insulin, TGF-β, AMPK, Ras, cell growth and metabolism. Conversely, the common TP downregulated genes participate in PI3K-Akt, Rap1, hippo, cAMP, apelin, p53, chemokine, B-cell receptor, NOD-like, PPAR, negative regulation of TGF-β, PDGFR, CD40, TLR5, Notch signaling pathways and processes like cell cycle, apoptosis, pyroptosis, somatic stem cell division, negative regulation of cell population proliferation and growth, activation of NF-kappaB-inducing kinase activity, tumour progression, cell motility and T-cell regulation (Supplementary Table. S3A-AC).

Furthermore, our examination on TR GBM unveiled that the upregulated genes unique to the cell types govern signaling pathways such as EGFR, MAPK, phospholipase C-activating G protein-coupled acetylcholine receptor, interleukin-7-mediated, regulation of neural precursor cell proliferation, negative regulation of T cell mediated cytotoxicity and replication. Contrariwise, the TR cell type unique downregulated genes are involved in ciliary neurotrophic factor-mediated, ionotropic glutamate receptor, mevalonate, BMP signaling pathways and mechanisms such as negative regulation of protein dephosphorylation, negative regulation of macroautophagy, pyruvate metabolism, cell cycle and positive regulation of neural precursor cell proliferation. Similarly, when we analyzed the mutual genes across cell types, we found that the common TR upregulated genes take part in Fas, NFκB, TORC2 signaling pathways and processes like tolerance induction to tumor cell, B-cell differentiation, cell growth, apoptosis, tumour suppression, pentose phosphate pathway and immune response. On the other hand, the common TR downregulated genes are involved in Notch, MAPK, NFκB signaling pathways and processes like carbon metabolism, glycolysis/gluconeogenesis, acetate metabolism and cellular migration and adhesion (Supplementary Table. S4A-S).

The engagement of these signaling pathways with GBM has been extensively studied [45], [46], [47], [48], [49], [50], [51], [52]. However, our analysis gives a holistic view of the mechanisms that are linked distinctively to the cell types and it unveils if the genes contributing to these molecular pathways are unique or common to the five cell types studied. There is growing evidence that inhibiting TGF-β signaling might give novel treatment options for GBM where TGF-β promotes its proliferation and survival [53]**.**

Our next approach consisted of studying the transcription factors (TFs) that monitor the above-mentioned genes. We mined the TFs from the Human TF atlas [25]. This disclosed TP and TR TFs that are unique and common to the cell types and are overseeing the expression of glioma genes (Fig. 5A-B) (Supplementary Tables 5A-T and 6A-H).

The TFs found in TR astrocyte, SIX3 has been discovered to suppresses glioblastoma cell growth and invasion via the WNT pathway [54], [55], [56] whereas the TF OVOL1 has been found to participate in EMT in cancers such as breast and colon [57]. NFKB2 TF found in TR microglia corroborates with recent studies that indicate NF-kB activation as a key cause of the malignant phenotype that leads to a poor prognosis in GBM patients [58]. TF BCL6 in OPC has been uncovered to encourage glioma and also to be a promising target to treat this cancer [59]. In TR MO, the TF BACH1 has been detected to aggravate p53 and increase glioblastoma resistance to temozolomide [60]. The TF, CREB3L2, found in TR NFO has been found to take part in malignant glioma survival pathway [61]. SOX1 discovered in TR OPC and NFO has been studied to promote GSCs to proliferate and self-renew [62]. KLF6 whose reduction advances NFκB signaling in glioblastoma [63] is found to be the common TF between TR astrocyte and NFO. Finally, SOX10 has been observed to be the common TF across all three cell types of oligodendrocytes in TR GBM. Sox10 is expressed widely in gliomas and promotes gliomagenesis triggered by platelet-derived growth factor-B [44]. Nevertheless, further analysis of TR TFs was not implemented since they were less in number.

Given our findings in TP dataset, we conducted the TP TF co-regulatory network analysis (Supplementary Fig. S1**A-H)**. In TP, this sequestered out 9 (astrocyte), 11 (microglia), 11 (MO), 13 (NFO), 16 (OPC & NFO), 8 (NFO & MO), 8 (OPC, NFO & MO) and 4 (Astrocyte, OPC & NFO) experimentally validated TFs, thereby excluding out 6 (astrocyte), 2 (microglia), 4 (MO), 10 (NFO), 10 (OPC & NFO), 5 (NFO & MO), 3 (Astrocyte, OPC & NFO) and 4 (OPC, NFO & MO) novel TFs, respectively (Supplementary Fig. S2**A-H)**. Further investigation of the TFs based on their correlation and TF influence activity revealed that the downregulated GLIS3 is the significant TF regulator in TP astrocyte. The increased expression of this factor has been studied to influence the glioma cells’ invasion, migration and proliferation activity, whereas its insufficient expression has an inhibitory effect on NF-κB signaling pathway [64]. In TP microglia, LTF, known to inhibit tumour cell growth [65], is the downregulated prominent TF monitor. In TP NFO, CENPA TF is also downregulated and found to be the chief TF governor. CENPA has been discovered to regulate the expression of key genes involved in cell proliferation, cell cycle, and centromere/kinetochore, and it also encourages tumor cell growth [66]. In TP MO, the principal TF governor was DMRTA2 which was found to be downregulated. DMRTA2 has been found to regulate the expression of Hes1 gene, and this gene has been studied to have roles in cancer stem cell (CSC) maintenance, metastasis, and drug-induced apoptosis antagonism [67], [68]. The notable TF regulating the common genes between NFO and MO in TP was revealed to be downregulated ASCL1. In a study, it was found that ASCL1 expression in a subgroup of GBM CSCs triggers neuronal target genes and enhances Notch inhibitor responsiveness, resulting in tumorigenicity being reduced [69]. Furthermore, in TP OPC and NFO, E2F7, which is found to be amplified in some tumours and controls the cell cycle by binding to RB1, is the downregulated eminent TF regulator [70]. The downregulated TEAD2 TF, which is common between all three types of TP oligodendrocytes, is the influential TF governer. This TF belongs to the TEAD family of TFs known to be necessary for development and have a crucial role in tumour initiation as well as progression [71]. Lastly, BCL11A TF is the prime upregulated TF governing the common genes between astrocyte, OPC and NFO in TP. It has been shown that BCL11A is linked to breast cancer cell carcinogenesis, proliferation, invasion, and metastasis by activating Wnt/-catenin signalling [72] (Fig. 6**A-H)**.

In addition, we conducted an in-depth study of the functional operations regulated by these TFs in their co-regulatory networks (Fig. 7**A-H)**. In TP astrocyte, we discovered that TFs ZNF878 and CREB3L4 are involved in highest number of processes such as signal transduction downstream of smoothened protein, activation of phospholipase C activity and peroxisome, positive regulation of translation, respectively. Phospholipase C (PLC) has been determined to control a range of cell functions such as cell motility, transformation, differentiation, and proliferation, and PLC also regulates cancer cells in part by serving as signaling intermediates for cytokines like EGF and interleukins [73]. In TP microglia, the principal regulator LTF is found to regulate TNF, TLR, chemokine, B-cell receptor signaling pathways and processes such as transcriptional misregulation, natural killer cell mediated cytotoxicity, phagocytosis and carbon metabolism in cancer. In addition, BATF TF is seen to regulate highest number of processes such as negative regulation of immune system, positive regulation of adaptive immune response and NFκB TF activity, B cell activation, T cell migration etc. In TP NFO, the significant TF regulator CENPA is involved in cell cycle whereas the third top influencer EN2 is involved in processes such as apoptosis. In TP MO, ASH1L is seen to regulate highest number of processes that include apoptosis, carbon metabolism in cancer and NFκB signaling pathway. In TP NFO and MO, SOX11, the second top TF influencer is observed to regulate signaling pathways such as TGF-β, MAPK, calcium, phosphatidylinositol and apelin, whereas TEAD3 is regulating highest number of processes like extrinsic apoptosis, NFκB pathway etc. In TP OPC and NFO, SP9 is seen to regulate highest number of processes significantly that include cAMP and MAPK signaling pathways. In TP OPC, NFO and MO, Suppression of Tumourigenicity 18 (ST18), regulating the highest number of processes, has been studied to regulate pro-inflammatory and pro-apoptotic gene expression [74]. The second notable TF regulator, DLX1, is involved in cGMP-PKG signaling pathway, and this cyclic GMP (cGMP)/protein Kinase G (PKG) pathway has been identified as an endogenous apoptotic mechanism in a variety of cancers, notably breast and colon cancers [75], [76], [77], [78], [79]. Lastly, in TP astrocyte, OPC and NFO, the TF SIX5 is found to be involved in the highest number of processes like regulation of GTPase activity, cell growth, complement activation, lectin pathway.

Once we had conducted our analysis at the transcriptomic level, we inspected the proteome activities undertaken in the GBM brain cells types individually. Given the importance of the association of domains in protein–protein interactions for a functional outcome to eventuate, we retrieved protein-domain interaction (PDI) data by logging the cell type-specific gene datasets onto the INstruct database (Supplementary Table S7A-X and S8A-I). We further added signal transduction information to these PDIs and visualized extensive protein-domain interaction networks (PDINs) using the SIGNOR database. Our investigation identified essential protein regulators and also uncovered the foundational pathways occurring due to these PDINs. In TP astrocyte, although the prime protein regulator could not be deduced, we found that the PDINs participate in neurotrophin signaling pathway and endocytosis (Fig. 8**A)**. In TP microglia, the significant protein regulators such as MAPK14 (domain- Pkinase) activates MAPKAPK3 (domain- Pkinase), binds to PXN, inhibits MAX (domain- HLH) and EIF4EBP1 (domain- eIF_4EBP). The next protein regulator NFKBIA (domain- Ank) inhibits NFKB1 and activates RELA (domain- RHD), whereas regulator BCL3 (domain- Ank) activates NFKB1 and indirectly binds to NFKB2 (domain- Ank). BCL3 is regulated by LTF, the prominent TF influencer detected in TP microglia. In addition, protein regulator SYK activates SH3BP2 and VAV1, and regulator TRADD (domain- Death) activates FADD (domain- Death) and RIPK1. Some fundamental signaling pathways like MAPK, regulation of pluripotency of stem cells and processes such as cytokine-cytokine receptor interaction, complement and coagulation cascades etc. are seen to be transpiring in this cell type (Fig. 8**B)**. The eminent protein regulators in TP NFO are CDK1 (domain- Pkinase) that activates CDC25A, inhibits WEE1 (domain- Pkinase), CASP9 (domain- Peptidase_C14), CASP8 (domain- Peptidase_C14), and regulator PTPN12 (domain- Y_phosphatase) that inhibits JAK2 (domain- Pkinase_Tyr), BCAR1 and PTK2B (domain- Pkinase_Tyr). These PDIs are observed to participate in essential processes like cell cycle, Rap1, PI3K-Akt signaling pathways, regulation of pluripotency of stem cells, complement and coagulation cascades (Fig. 8**D)**. In TP MO, the notable protein regulator was detected to be PDPK1 (domain- Pkinase) that activates PRKCE (domain- Pkinase) and PRKCA. This protein regulator is found to be governed by the prime TF influencer in this cell type i.e. DMRTA2 (Fig. 8**E)**. The PDI common between TP astrocyte and MO is involved in cell adhesion (Fig. 8**F)**. The PDI common between TP microglia and OPC participates in cell cycle (Fig. 8**G).** The significant protein regulators in the PDIN common between TP microglia and NFO are PRKCA (domain- Pkinase) that activates NCF1, CYBA, inhibits LCK (domain- SH3_1), binds to ITGB2, SNAP23, DGKZ (domain- C1_1), and regulator SYK that activates PRKCA (domain- Pkinase) and inhibits LCK (domain- SH3_1). This PDIN is engaged in NFκB, Rap1 signaling pathways and natural killer cell mediated cytotoxicity (Fig. 8**H)**. Whereas the PDI shared between TP OPC and MO participates in axon guidance (Fig. 8**I)**. The three influential protein regulators in PDIN common between TP OPC and NFO are SRC that activates EPHA2 (domain- Ephrin_Ibd), IGF1R, LRP1, DLG4, JUP, CTNNB1 (domain- Arm), CDH5, RAC1 (Ras), TIAM1 (domain-PH), inhibits ITGAL and binds to GRB2, MMP14, PTPN12. Followed by PDGFRB that binds to GRB2, SRC and FYN, and lastly, PLK1 (domain- Pkinase) that activates CHEK2 (domain- Pkinase), binds to CTNNB1 (domain- Arm) and BRCA2 (domain- BRCA2). PLK1 regulator has been found to be regulated by the chief TF influencer detected i.e. E2F7. This PDIN is associated with vital signaling pathways such as JAK-STAT, PI3K-Akt, cAMP, apelin, AGE-RAGE, hippo and processes such as cell cycle, transcriptional misregulation in cancer, fluid sheer stress (Fig. 8**J)**. The PDIs mutual between TP NFO and MO are engaged in TNF, Rap1, Ras, Phospholipase D signaling pathways and process such as natural killer cell mediated cytotoxicity (Fig. 8**K)**. Whereas the PDIs common between TP astrocyte, OPC and NFO are involved in Notch, AMPK signaling pathways (Fig. 8**L)**. We found notch, AMPK signaling pathways regulated in astrocyte, OPC and NFO in TP (Fig. 8**M)**. In TP microglia, NFO and MO, the chief protein regulator was recognized as LCK (domain- Pkinase_Tyr) that activates PRKCD and PTPN6 (domain- SH2). This PDIN is witnessed to take part in glioma, Rap1 and T-cell receptor signaling pathways (Fig. 8**N)**. Finally, in the PDIN shared between all the three cell types of oligodendrocytes, the principal protein regulator is inferred to be CDK2 (domain- Pkinase) that activates UBE2A, MCM2, MCM3, and inhibits CDKN1A (domain- CDI), TPX2 (domain- Aurora-A_bind). Cell cycle and p53 signaling pathway are the crucial processes affiliated to this PDIN (Fig. 8**O).** Correspondingly, the SIGNOR data was available for only two TR subsets of proteins. In TR NFO, the PDI is observed to be involved in T-cell receptor signaling pathway (Fig. 9**B)**.

A similar analysis of TP and TR proteins was performed that lacked the SIGNOR visualization and regulation data (Fig. 10**A-B)**. The following functional processes are seen to be operated by the TP proteins in the cell types: invasive growth, cell migration, signal transduction inhibition, apoptosis, oncogenesis, cell cycle, autophagy, cell attachment/migration/differentiation, and complement system. Analogously, the processes driven by the TR proteins in cell types are: apoptosis, T cell activation/homeostasis/proliferation, cell growth/survival/adhesion/motility, phagocytosis, cell cycle arrest, ubiquitination, signaling pathways like TNF, NFκB, PI3k, PDGFR and Wnt. In sum, our analysis gives a comprehensive and wider perspective of the various phenomena that emerge in the GBM cell types.

## 5 Conclusion

In this study, we examined the brain cell types in GBM. Astrocyte, microglia, OPC, NFO and MO specific TP and TR glioma genes were extracted from the openly accessible transcriptome dataset of GBM. We studied the GBM grade 4 gene expression in each of the glial cell types and performed comprehensive network analyses to understand cell type specific pathways. This underlined cell type specific prominent transcription factors regulating significant pathways and also identified prime protein regulators in protein-domain interaction networks. Furthermore, we observed that the chief TF influencers, LTF, DMRTA2 and E2F7, govern the eminent protein regulators BCL3, PDPK1 and PLK1, respectively. Some distinctive as well as mutual processes and functional pathways have been deduced to take place in these GBM borne glial cell types. Our study opens avenue for inspection and modulation of these mechanisms in the brain cell types in order to refine the therapeutic routes to treat this aggressive cancer.

## CRediT authorship contribution statement

**Samreen Fathima:** Software, Data curation, Investigation, Resources, Formal analysis, Writing- original draft, Writing- review & editing. **Swati Sinha:** Formal analysis, Supervision, Project administration, Writing – review & editing. **Sainitin Donakonda:** Conceptualization, Software, Methodology, Project administration, Supervision, Formal analysis, Writing – original draft, Writing – review & editing.

## Declaration of Competing Interest

The authors declare that they have no known competing financial interests or personal relationships that could have appeared to influence the work reported in this paper.
