# Supplementary material for: Unraveling unique and common cell type-specific mechanisms in glioblastoma multiforme
Source: Comput Struct Biotechnol J. 2021 Dec 9;20:90–106. doi: 10.1016/j.csbj.2021.12.010 (PMC8688884; doi:10.1016/j.csbj.2021.12.010)
Supplement: Supplementary data 1 [file mmc1.docx]

**Supplementary Data**

**Unraveling Unique and Common Cell Type-Specific Mechanisms in Glioblastoma Multiforme**

Samreen Fathima^1^, Swati Sinha^1^, Sainitin Donakonda^2#^

1. Department of Biotechnology, Faculty of Life and Allied Health Sciences, MS Ramaiah University of Applied Sciences, Bangalore,India

2. Institute of Molecular Immunology and Experimental Oncology, Klinikum Rechts Der Isar, Technical University of Munich, Munich, Germany

**^#^** Corresponding author

Sainitin Donakonda Ph.D: [sainitin.donakonda@tum.de](mailto:sainitin.donakonda@tum.de)

Institute of Molecular Immunology and Experimental Oncology, Klinikum Rechts Der Isar, Technical University of Munich, Munich, Germany, Tel: +4917674167311,

ORCID ID: orcid.org/0000-0003-3216-8759.

**Supplementary Figure 1**

**Figure S1.** Transcription factor co-regulatory networks in TP glioma cell types. (**A-H**) Co-regulatory networks of experimentally validated TFs in astrocyte (n= 8), microglia (n=10), MO (n=11), NFO (n=13), OPC&NFO (n=16), NFO&MO (n=8), astrocyte&OPC&NFO (n=4), and OPC&NFO&MO (n=8) were visualized in Cytoscape v3.8.0. The diamond represents TFs and the circle represents genes. Note: Red and blue color denote up and down regulation, respectively.

**Supplementary Figure 2**

**Figure S2.** Novel transcription factor correlation networks in TP glioma cell types. (**A-C**) The correlation networks visualized in Cytoscape v3.8.0 depict novel TFs in astrocyte (n= 6), microglia (n=2), MO (n=4) and NFO (n=10), OPC&NFO (n=10), NFO&MO (n=5), astrocyte&OPC&NFO (n=3), and OP&NFO&MO (n=4) and Note: Red and blue color denotes up and down regulation, respectively.
