# Supplementary material for: Unraveling unique and common cell type-specific mechanisms in glioblastoma multiforme
Source: Comput Struct Biotechnol J. 2021 Dec 9;20:90–106. doi: 10.1016/j.csbj.2021.12.010 (PMC8688884; doi:10.1016/j.csbj.2021.12.010)
Supplement: Supplementary data 2 [file mmc2.pdf]

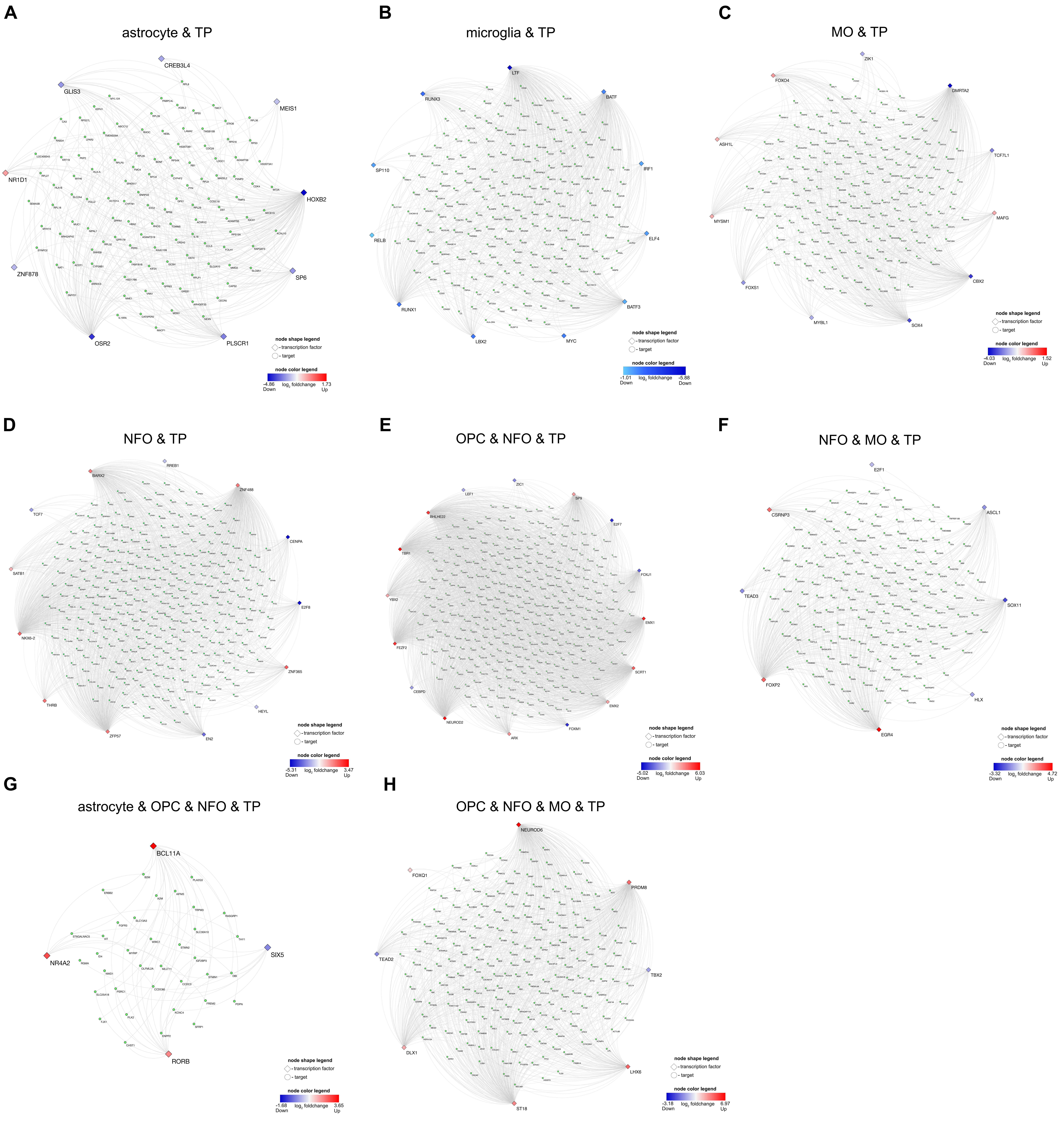
A
astrocyte & TP
CREB3L4
GLIS3
MEIS1
NR1D1
HOXB2
ZNF878
SP6
OSR2
PLSCR1
node shape legend
- transcription factor
- target
node color legend
-4.86 Down
log2 foldchange
1.73 Up
B
microglia & TP
LTF
RUNX3
BATF
SP110
IRF1
RELB
ELF4
RUNX1
BATF3
LBX2
MYC
node shape legend
- transcription factor
- target
node color legend
-1.01 Down
log2 foldchange
-5.88 Down
C
MO & TP
ZIK1
FOXO4
DMRTA2
ASH1L
TCF7L1
MYSM1
MAFG
FOXS1
CBX2
MYBL1
SOX4
node shape legend
- transcription factor
- target
node color legend
-4.03 Down
log2 foldchange
1.52 Up
D
NFO & TP
RREB1
BARX2
ZNF488
TCF7
CENPA
SATB1
E2F8
NKX6-2
ZNF365
THRB
HEYL
ZFP57
EN2
node shape legend
- transcription factor
- target
node color legend
-5.31 Down
log2 foldchange
3.47 Up
E
OPC & NFO & TP
ZIC1
LEF1
SP9
BHLHE22
E2F7
TBR1
FOXJ1
YBX2
EMX1
FEZF2
SCRT1
CEBPD
EMX2
NEUROD2
FOXM1
ARX
node shape legend
- transcription factor
- target
node color legend
-5.02 Down
log2 foldchange
6.03 Up
F
NFO & MO & TP
E2F1
CSRNP3
ASCL1
TEAD3
SOX11
FOXP2
HLX
EGR4
node shape legend
- transcription factor
- target
node color legend
-3.32 Down
log2 foldchange
4.72 Up
G
astrocyte & OPC & NFO & TP
BCL11A
NR4A2
SIX5
RORB
node shape legend
- transcription factor
- target
node color legend
-1.68 Down
log2 foldchange
3.65 Up
H
OPC & NFO & MO & TP
NEUROD6
FOXQ1
PRDM8
TEAD2
TBX2
DLX1
LHX6
ST18
node shape legend
- transcription factor
- target
node color legend
-3.18 Down
log2 foldchange
6.97 Up
