# Supplementary figures and images for: Unraveling unique and common cell type-specific mechanisms in glioblastoma multiforme
Source: Comput Struct Biotechnol J. 2021 Dec 9;20:90–106. doi: 10.1016/j.csbj.2021.12.010 (PMC8688884; doi:10.1016/j.csbj.2021.12.010)

**A**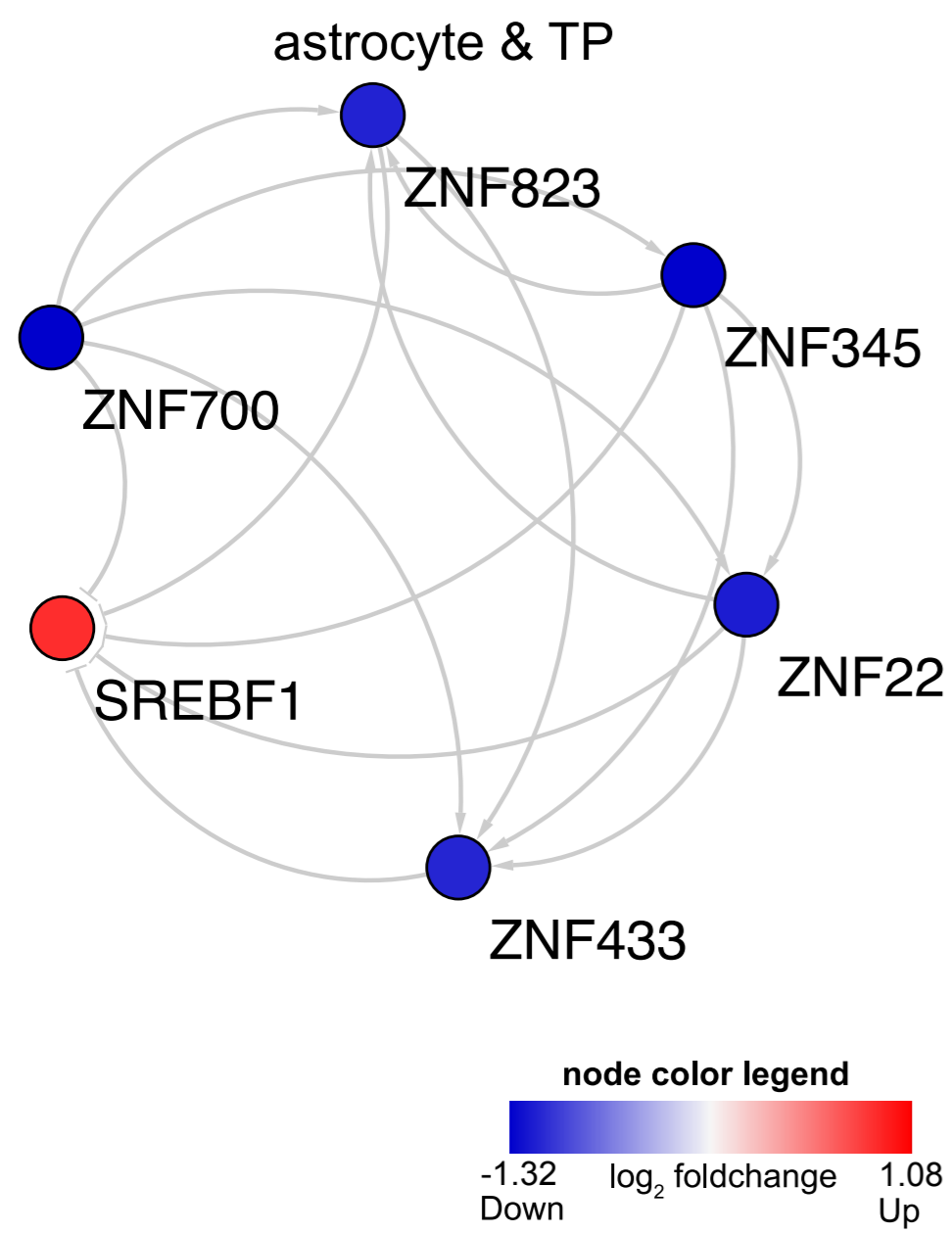**B**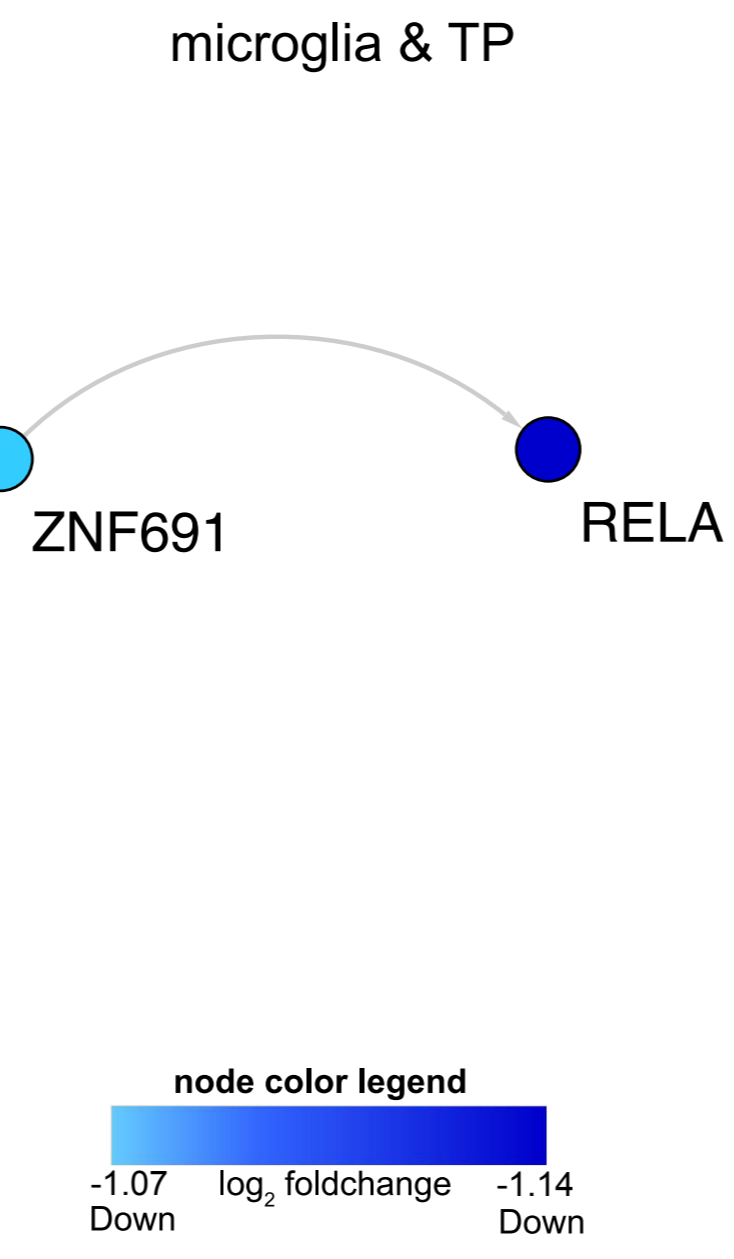**C**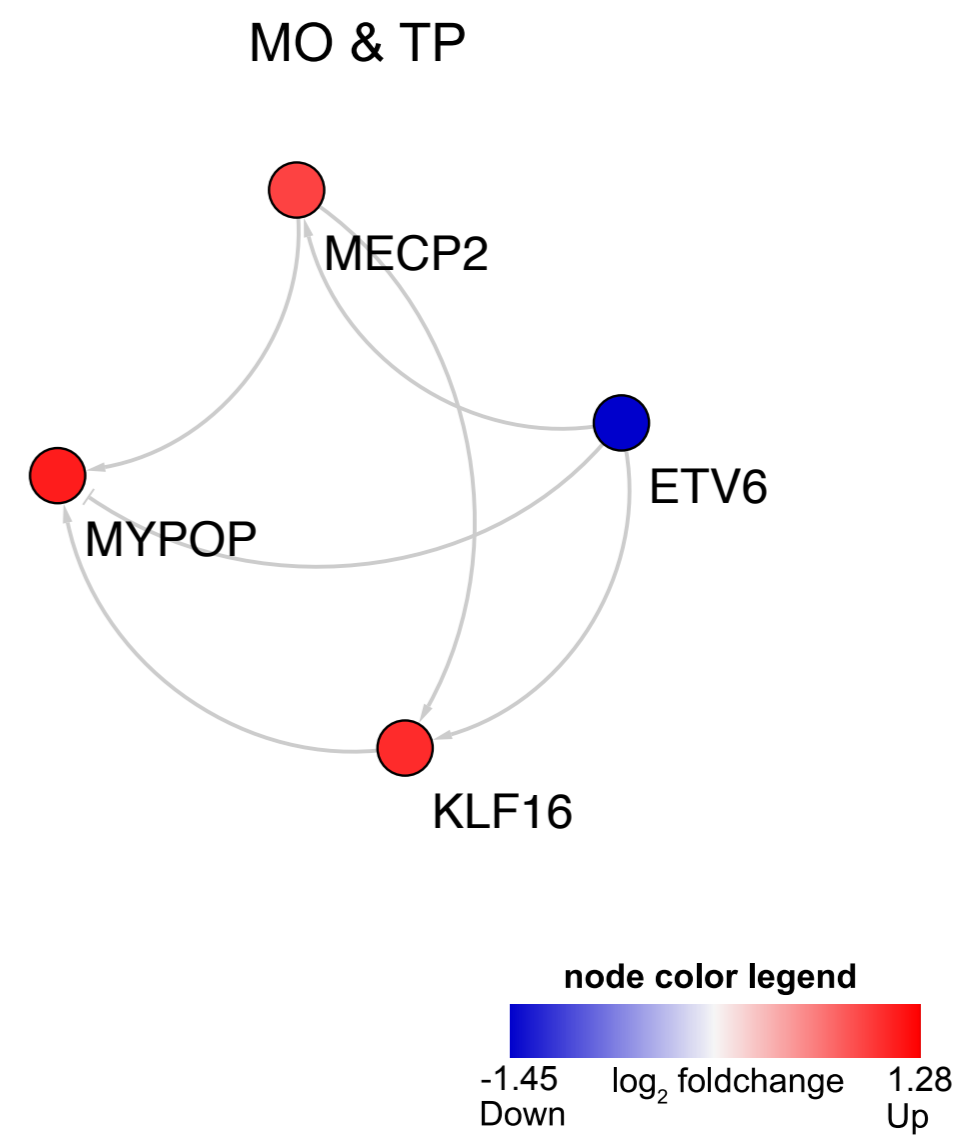**D**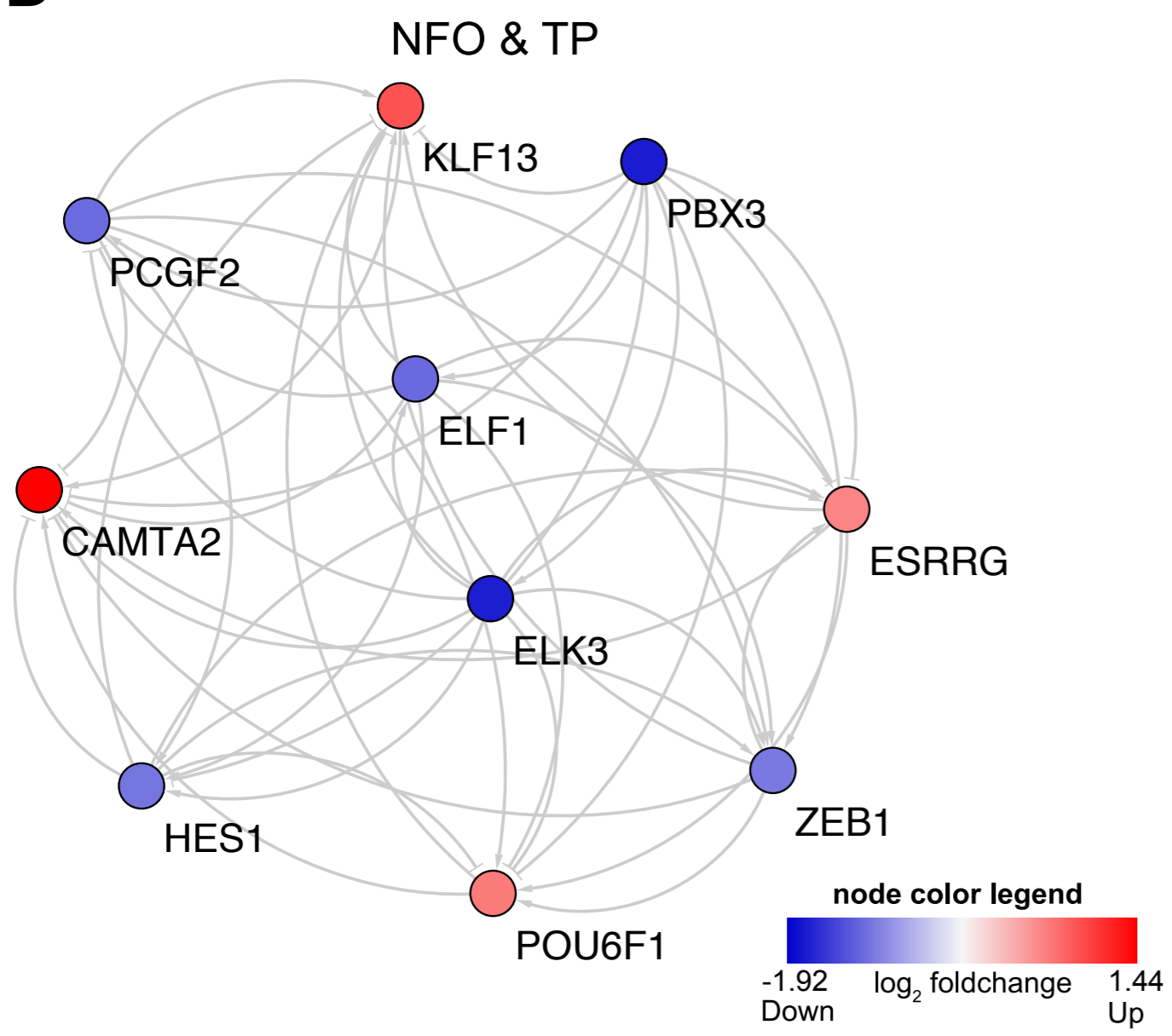**E**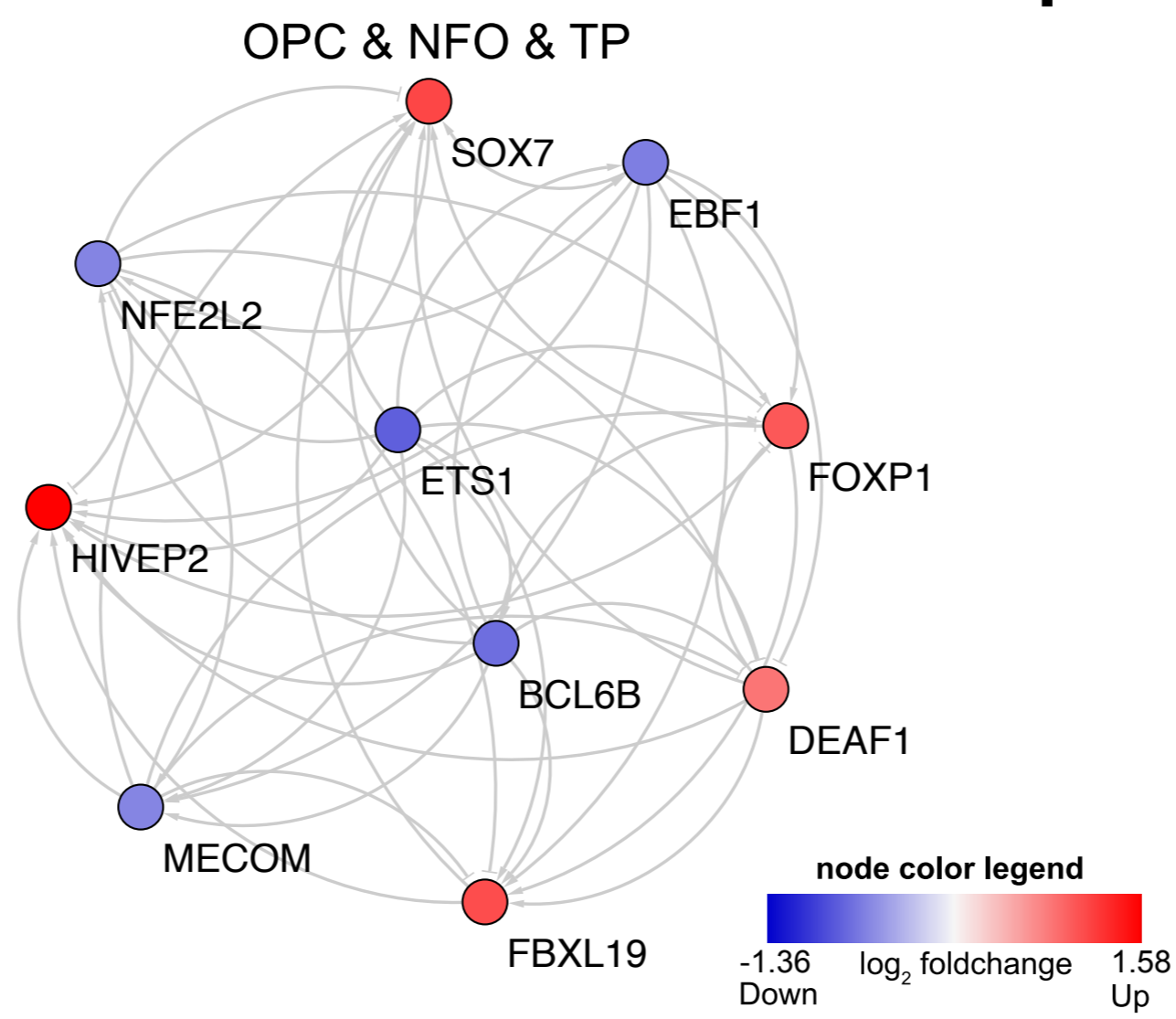**F**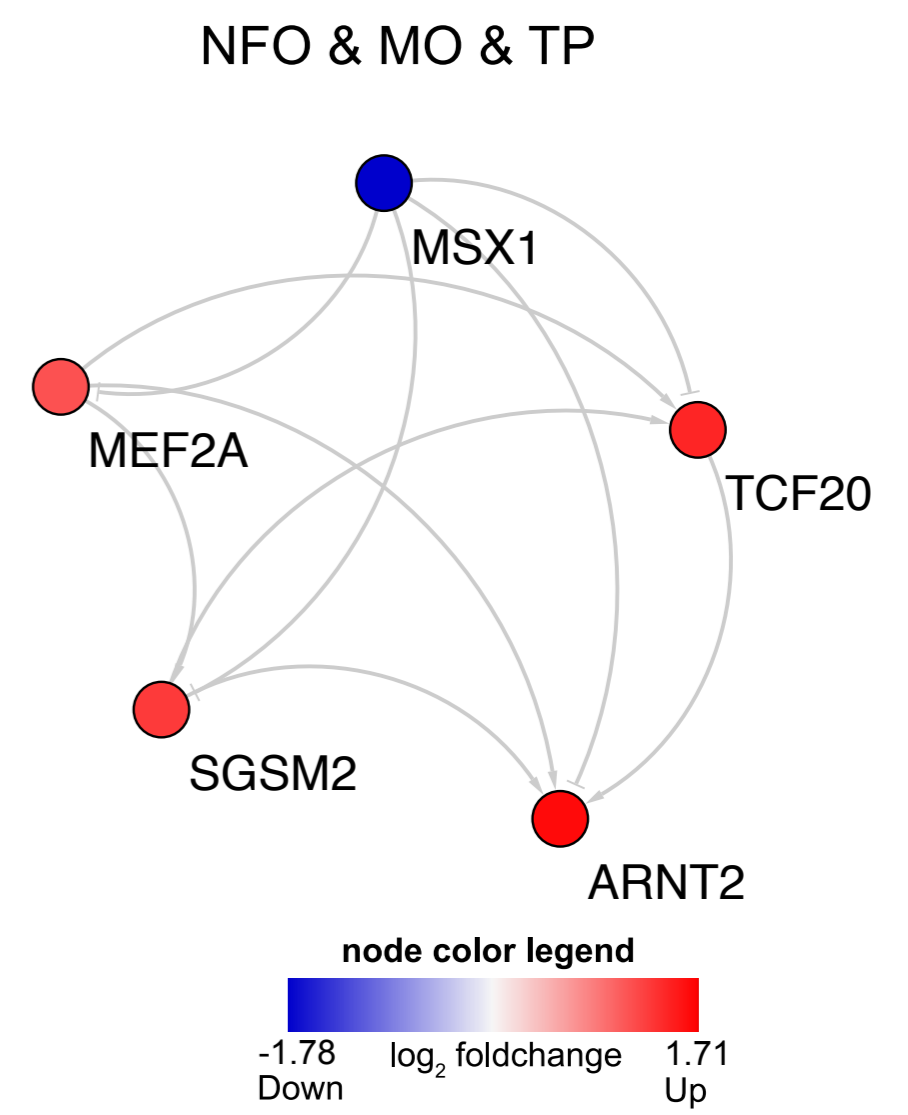**G**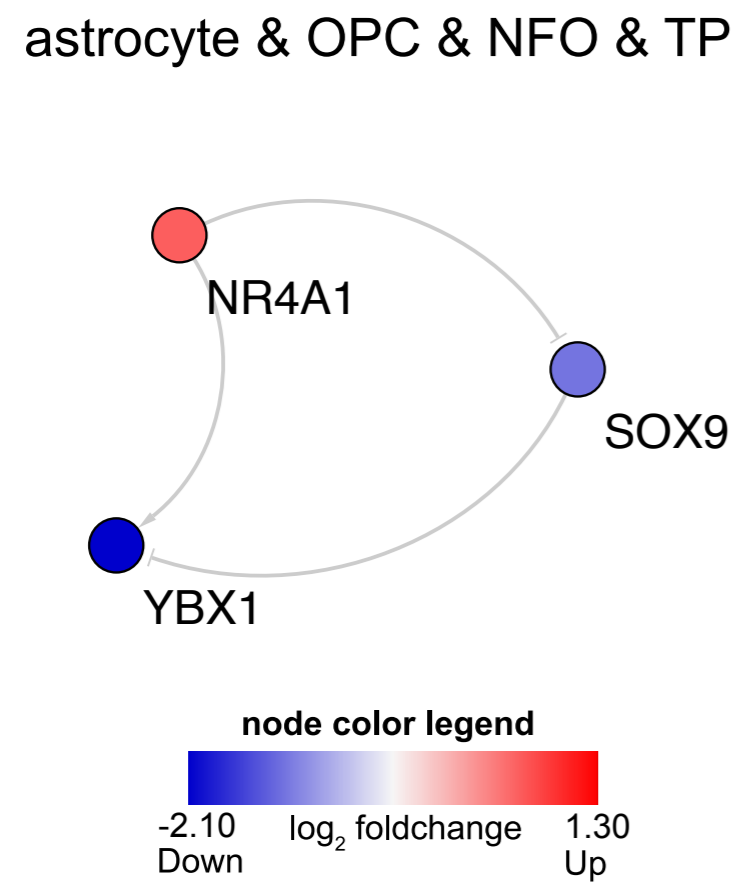**H**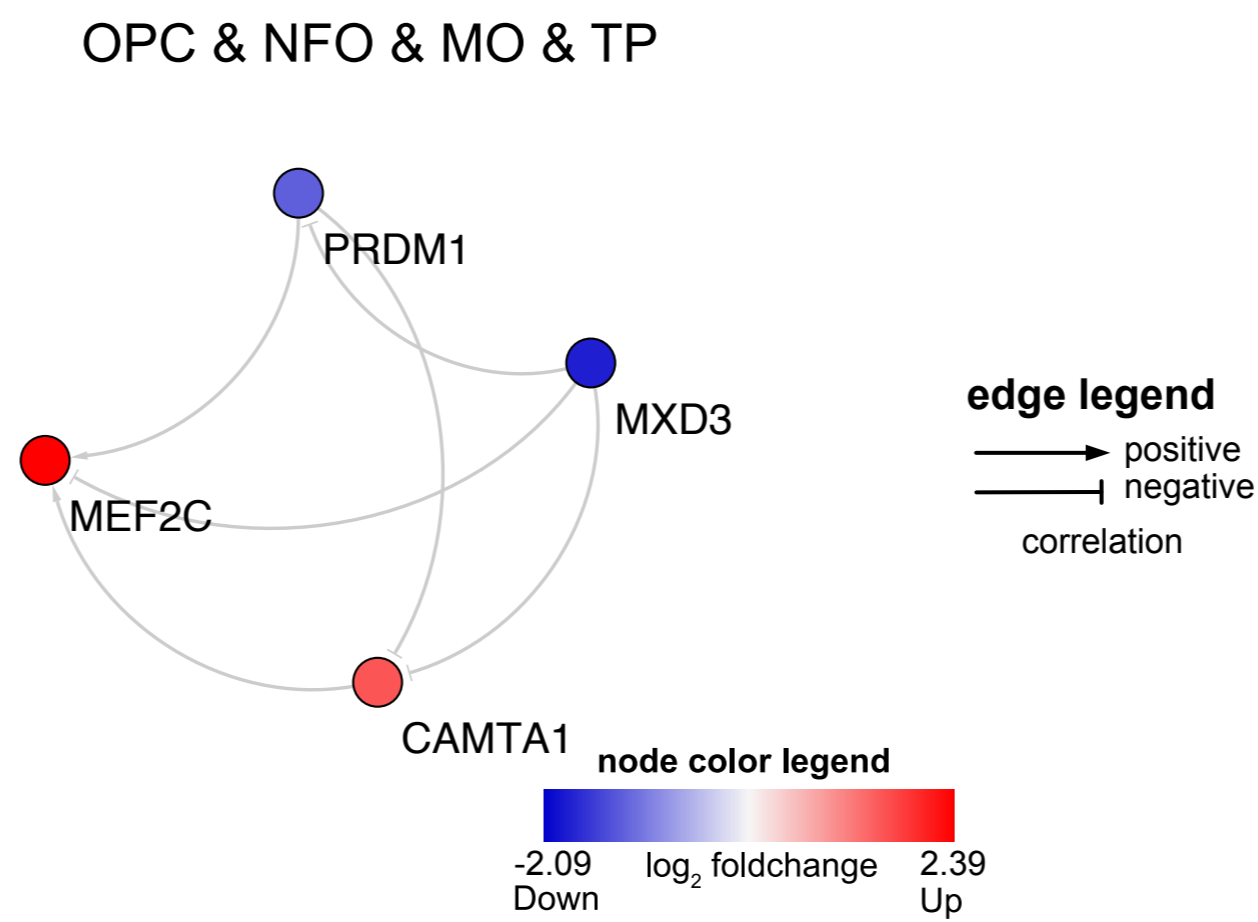

Supplement: Supplementary data 3 [file mmc3.pdf]
